# Reinforcing Tunnel Network Exploration in Proteins Using Gaussian Accelerated Molecular Dynamics

**DOI:** 10.1021/acs.jcim.4c00966

**Published:** 2024-08-15

**Authors:** Nishita Mandal, Bartlomiej Surpeta, Jan Brezovsky

**Affiliations:** † Laboratory of Biomolecular Interactions and Transport, Department of Gene Expression, Institute of Molecular Biology and Biotechnology, Faculty of Biology, Adam Mickiewicz University, Uniwersytetu Poznanskiego 6, Poznan 61-614, Poland; ‡ International Institute of Molecular and Cell Biology in Warsaw, Ks Trojdena 4, Warsaw 02-109, Poland

## Abstract

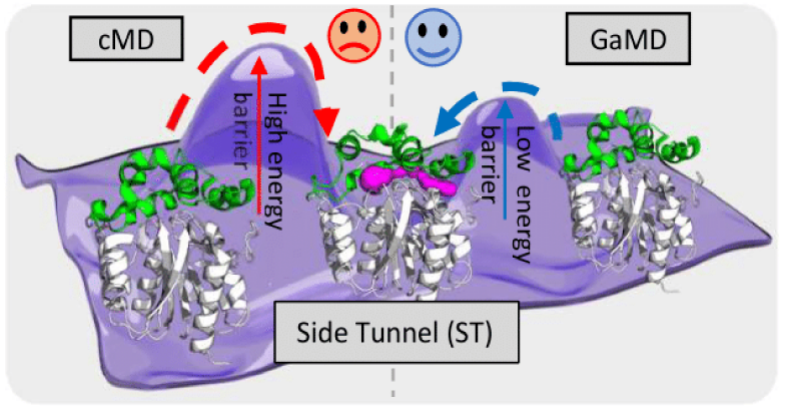

Tunnels are structural conduits in biomolecules responsible for transporting chemical compounds and solvent molecules from the active site. They have been shown to be present in a wide variety of enzymes across all functional and structural classes. However, the study of such pathways is experimentally challenging, because they are typically transient. Computational methods, such as molecular dynamics (MD) simulations, have been successfully proposed to explore tunnels. Conventional MD (cMD) provides structural details to characterize tunnels but suffers from sampling limitations to capture rare tunnel openings on longer time scales. Therefore, in this study, we explored the potential of Gaussian accelerated MD (GaMD) simulations to improve the exploration of complex tunnel networks in enzymes. We used the haloalkane dehalogenase LinB and its two variants with engineered transport pathways, which are not only well-known for their application potential but have also been extensively studied experimentally and computationally regarding their tunnel networks and their importance in multistep catalytic reactions. Our study demonstrates that GaMD efficiently improves tunnel sampling and allows the identification of all known tunnels for LinB and its two mutants. Furthermore, the improved sampling provided insight into a previously unknown transient side tunnel (ST). The extensive conformational landscape explored by GaMD simulations allowed us to investigate in detail the mechanism of ST opening. We determined variant-specific dynamic properties of ST opening, which were previously inaccessible due to limited sampling of cMD. Our comprehensive analysis supports multiple indicators of the functional relevance of the ST, emphasizing its potential significance beyond structural considerations. In conclusion, our research proves that the GaMD method can overcome the sampling limitations of cMD for the effective study of tunnels in enzymes, providing further means for identifying rare tunnels in enzymes with the potential for drug development, precision medicine, and rational protein engineering.

## Introduction

Enzymes function as natural catalysts to enable the execution of complex reactions in living cells. However, the structural basis of their efficiency and sensitivity remains incompletely understood due to the complexity of their specific biochemical reactions. Enzymatic reactions occur at an active site, which may be localized either on their surfaces, in pockets that are generally surface accessible, or in cavities buried within the protein’s core.^1,2^ In enzymes with buried active sites, the transport of molecules from the bulk solvent to the active site cavity and their release is carried out through transport pathways known as tunnels,^2−7^ sometimes called channels.^8−10^ Enzymes often feature multiple tunnels, some of which are dedicated to transporting particular molecules.^6,11−13^ Such tunnels are formed from separate continuous voids in biomolecular structures. Their narrow parts, known as bottlenecks, often determine the selectivity for compounds passing through or out of the active site cavity. Bottlenecks are primarily controlled through gates represented by residues, loops, secondary structures, or domains.^14^ Due to the presence of these gates, the tunnels are transient, making their characterization a nontrivial task and sometimes even impossible in a single static structure.^6,14,15^ Gates are frequently involved in regulating transport, especially in multistep reactions.^14,15^ Gating residues often act as selectivity filters, allowing only certain-sized substrates or products to traverse the tunnel, thereby playing a crucial role in the catalytic activity of the enzyme.^14^

These tunnels are widespread, comprising over 50% of enzymes across all six Enzyme Commission (EC) classes.^1^ To understand the structural basis of activity related to these tunnels, in-depth investigation is required to study the dynamic behavior of these pathways, which determines the exchange rate of substrates entering the active site or products being released into bulk solvents.^6^ The tunnels also regulate the movement of solvents and products,^15^ providing additional control over enzymatic reactions. Given that many enzymes containing molecular tunnels have been associated with various diseases and that the inhibitors binding to these tunnels can function as effective medications, we can comprehend the biological significance of tunnels.^15^ Mutations in tunnel residues can result in variants with significantly altered properties.^3,16^ Recent research shows that the catalytic functions of enzymes and their prospects can be altered by the dynamics, geometry, and physicochemical properties of the tunnel.^15^ Therefore, the dynamics and flexibility of enzymes and their tunnels must be considered important factors when studying structural biology in the context of structure-based drug design.^15^

Due to advancements in experimental methods such as X-ray crystallography,^17^ cryo-EM,^18^ various types of NMR spectroscopy,^19^ and cutting-edge computational technologies such as deep learning-based protein structure prediction with AlphaFold,^20^ an increasing number of high-quality three-dimensional static structures are available for in-depth investigation, including the analysis of potential tunnels. Although experimental methods yield the structures of proteins, the study of tunnels within these structures requires dedicated geometry-based tools that explore free van der Waals volume, such as CAVER 3.0,^21^ MOLE 2.5,^22^ and MolAxis.^23^ Because static structures derived from experimental techniques or computational predictions usually do not consider multiple protein conformational states, these techniques are often followed by molecular dynamics (MD) simulations to explore the protein conformational landscape and capture tunnel dynamics, thereby studying their transient nature.^15,17^ Additionally, the exploration of tunnels by geometry-based methods can be supplemented by ligand-tracking methods using tools such as streamline tracing,^24^ Visual Abstraction of Solvent Pathlines,^25^ Watergate,^26^ AQUA-DUCT,^27^ and trj_cavity.^28^

Due to the difficulty of biomolecules easily overcoming large energy barriers, milliseconds to seconds or even longer sampling is required to visit rare structural changes.^17,29^ The opening of transient tunnels poses a significant challenge for conventional MD (cMD) methods, which are typically limited to tens of microseconds.^30^ To address this limitation, various biased enhanced sampling methods have been proposed, such as adaptive biasing force (ABF),^31^ umbrella sampling,^32^ and metadynamics,^33^ which have been successfully used to deliver considerable insights into the utilization of particular pathways by selected small molecules.^34−42^ However, their main limitation lies in defining the collective variables (CVs) capable of reaching sufficient convergence even using very intensive computation,^43−45^ since this requires thorough knowledge of the system and often extensive optimization to find an acceptable solution.^46−49^ For systems with complex tunnel networks, multiple CVs would be required to explore the desired transport pathways efficiently. Alternatively, methods that enhance ligand movements more stochastically, without the need for a rigorous definition of transport pathways, like random accelerated MD (RAMD),^50,51^ accelerated MD (aMD),^52^ or ligand Gaussian accelerated MD (LiGaMD),^53,54^ face a challenge in the identification of rarer transport pathways as most simulation replicates tend to explore the primary pathways.^16,43,55−57^ Moreover, the obtained information on tunnel usage is usually restricted to the particular ligand explicitly used in the simulations, which cannot always be easily translated to different ligands with different molecular properties.^58,59^ Hence, efficient exploration of tunnel conformational dynamics directly from the protein structure without explicitly probing and thus biasing them with particular ligands remains challenging. From this perspective, aMD and Gaussian accelerated MD (GaMD),^60^ which have been proposed to enhance the sampling of conformational space without explicitly defined CVs,^52^ represent a promising solution for tunnel exploration. Furthermore, GaMD provides an opportunity to reconstruct the original free energy landscape due to the appropriately controlled boosting potential. Although GaMD has been investigated for numerous enzymes, including explicit ligand migration simulations,^61^ its effectiveness for reliably sampling conformations of an overall tunnel network in the absence of ligands remains unknown.

**Figure 1:**
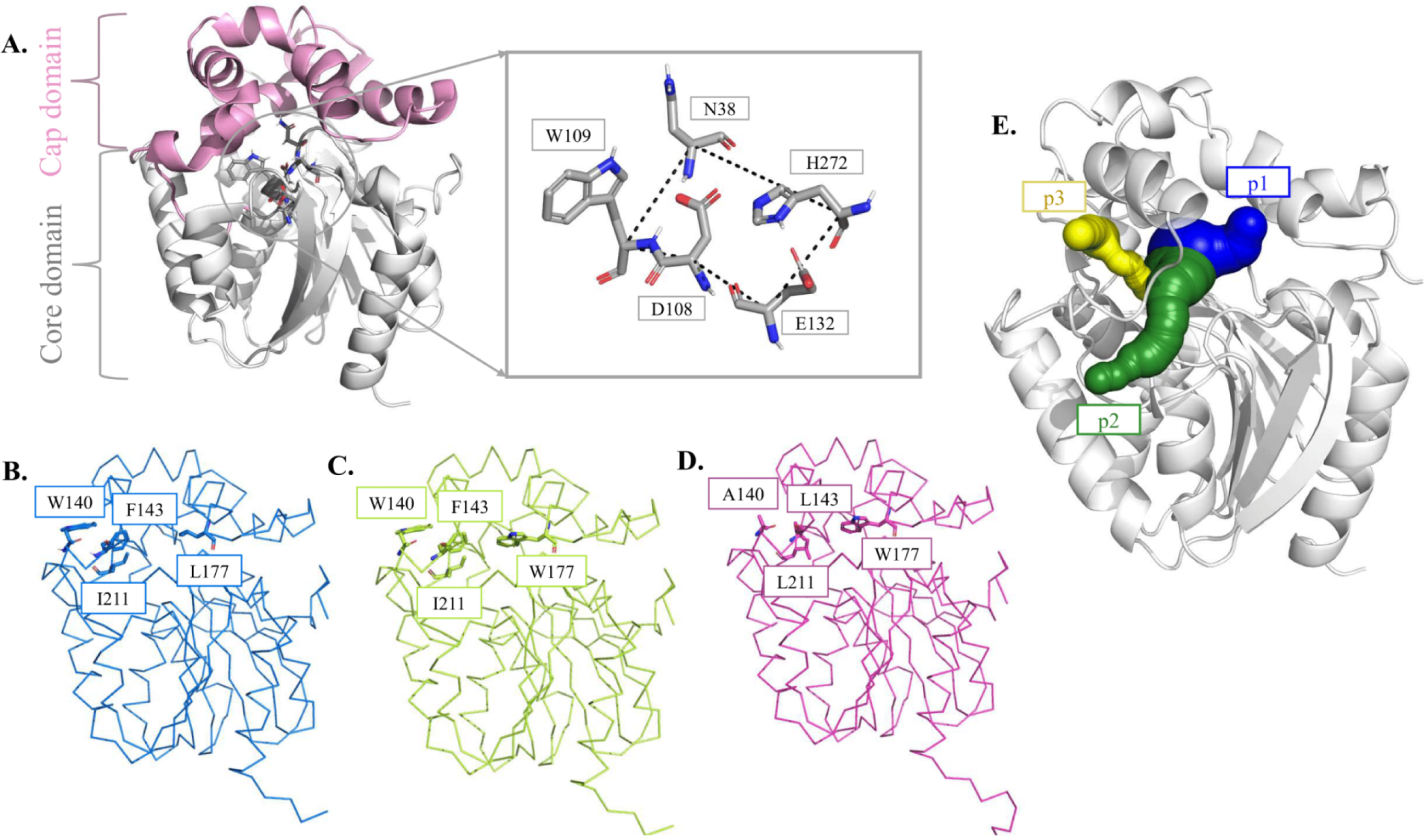
Structural representations of LinB and its variants. A. LinB has a more flexible cap domain (pink cartoon) and a more stable core domain (gray cartoon). The catalytic residues of LinB forming the catalytic pentad are shown as gray sticks. B. LinB-Wt. C. LinB-Closed mutant carrying the L177W mutation. D. LinB-Open mutant with four mutations: L177W, F143L, W140A, and I211L. E. Known p1, p2, and p3 tunnels of LinB. The shown representative tunnels were selected from GaMD simulations of LinB-Wt to have their bottleneck radius and length match the average properties of individual tunnel clusters observed in these simulations.

In this study, we examine the efficiency of GaMD to explore stable and transient tunnels in haloalkane dehalogenase’s (LinB) tunnel network. LinB belongs to the α/β hydrolase superfamily, which catalyzes the hydrolytic dehalogenation of halogenated compounds,^62^ with high potential applications in bioremediation, biosensing, or biocatalysis.^63,64^ Importantly, due to its multistep reaction and utilization of water during the reaction, this class of enzymes requires multiple tunnels crucial for synchronizing particular steps, making it a perfect model for our investigation.^65^ Structurally, LinB has a flexible cap domain (Figure 1A) and stable core domain,^62^ and its active site consists of a catalytic pentad (E132–a catalytic acid, D108–a nucleophile, H272–a base, W109 and N38–two halide-stabilizing residues).^66^ LinB has three known tunnels: one permanent tunnel (p1) and two auxiliary tunnels (p2 and p3).^16^ Therefore, in the present study, we focused on three variants to thoroughly test GaMD capabilities to explore the conformational space of the known LinB tunnel network. These variants include (i) LinB wild-type (LinB-Wt), in which p1 represents the main tunnel, whereas p2 tunnels play an auxiliary role (Figure 1B); (ii) designed LinB-Closed variant carrying the L177W mutation resulting in a drop of p1 tunnel occurrence (Figure 1C);^16^ and (iii) LinB-Open variant, in which the main p1 tunnel was closed by the same mutation L177W as seen in the former variant, resulting in a reduction in p1 functioning as the main tunnel. Introduced mutations W140A, F143L, and I211L result in the opening of an additional auxiliary p3 tunnel (Figure 1D).^16^ Given that the tunnel dynamics and function vary significantly across the selected LinB variants, they represent suitable models for the exploration of GaMD capabilities to capture rare tunnel dynamics, validate its efficiency and sensitivity with the broad knowledge from the literature, and compare it with cMD simulations.

## Methods

### System Setup and Conventional Molecular Dynamics Simulations

The cMD simulation was performed on three LinB variants: LinB-Wt (PDB code: 1MJ5),^62^ LinB-Closed (PDB code: 4WDQ),^16^ and LinB-Open (PDB code: 5LKA).^16^ First, the systems were protonated using the H++ server^67^ at pH 8.5^16^ and a salinity of 0.1 M. Subsequently, the systems were solvated using 4-point OPC water models^68^ and neutralized with counterions (Na^+^ and Cl^–^) to achieve a final NaCl concentration of 0.1 M. Initially, the systems were energy minimized using 500 steps of steepest descent, followed by 500 steps of conjugate gradient with decreasing harmonic restraints in 5 rounds using the PMEMD^69^ module of AMBER18^79^ with the ff14SB force field.^71^ The following restraints were applied to the system: 500 kcal  on all heavy atoms of the enzyme, followed by 500, 125, 25, and 0 kcal  on backbone atoms of the enzyme exclusively. Minimization was followed by a 2 ns equilibration MD simulation, with gradual heating to 310 K under a constant volume using the Langevin thermostat^72^ with a collision frequency of 1.0 ps^–1^ and harmonic restraints of 5.0 kcal  on the positions of all enzyme atoms, using periodic boundary conditions with the particle mesh Ewald method.^73,74^ The Berendsen barostat was used to control the pressure of the system. The simulations were run using a 4 fs time step enabled by SHAKE and the hydrogen mass repartitioning algorithm.^75^ Finally, these simulations were continued with an unrestrained 200 ns production simulation performed with pmemd.cuda at constant pressure and temperature, with frames stored every 20 ps.

Clustering analysis was performed using the hierarchical agglomerative (HierAgglo) algorithm in cpptraj^76^ based on a 200 ns trajectory for each of the three systems. A cutoff of 4.5 was used, with a number of clusters set to 5 and the linkage method as the average linkage, to obtain five clusters of the most diverse conformations. For each cluster, the frame with the lowest cumulative distance to all other frames within the cluster was selected as its representative. These five frames were used as seed structures for the production cMD, which continued to an unrestrained 5 μs simulation, with frames stored every 200 ps, under constant pressure and temperature for all three LinB variants.

### Gaussian Accelerated Molecular Dynamics

The five most diverse structures obtained by clustering analysis in cMD were used as seeds for GaMD in all three LinB variants to diversify the sampling space. After preparing the structures for GaMD simulation, a short 8 ns cMD was run to collect the potential energy statistics of the system, including maximum (*V*_max_), minimum (*V*_min_), average (*V*_av_), and standard deviation (σ*V*).^60^ GaMD equilibration of 8 ns was performed after adding the boost potential.^60^ The first 16 ns of each run was further considered as equilibration and excluded from the final analyses. Parameters governing the strength of the applied boosting potential were set as follows: σ0P, which is the standard deviation of the first potential boost on the total potential energy of the system, was set to 1.3 kcal/mol; σ0D, which is the standard deviation of the second potential boost on the dihedral angle of the system, was set to 2.5 kcal/mol. These parameters for σ0P and σ0D were tested iteratively using a range of values to bring the coefficients *k*0D and *k*0P as close as possible to a maximum value of 1.0, which provides the highest acceleration to the system, or the highest possible value whenever the system was unstable before reaching the maximum. The parameters used for testing are described in detail in Table S1. The system threshold energy was set to the lower bound (*E* = *V*_*max*_). Simulations were run using a 4 fs time step, analogously to cMD, with hydrogen mass repartitioning applied.^75^ Finally, these dual-boost GaMD simulations were continued for 5 μs of unconstrained production MD. The simulations were performed analogously to cMD settings, namely, under constant pressure and temperature using AMBER18 with the ff14SB force field, with frames stored every 200 ps for subsequent analyses.

### Basic Analysis

Trajectories generated from both methods, for all three LinB variants and corresponding replicates (totaling 30 repetitions of 5 μs sampling), were processed using cpptraj^70^ implemented in AmberTools17.^67^ The root-mean-square deviation (RMSD) and root-mean-square fluctuation (RMSF) were calculated with the initial structure as a reference, considering the backbone heavy atoms of the protein (N, CA, and C). RMSD calculations excluded the N-terminal tail (comprising the first 11 amino acids) due to its high overall flexibility. The protein’s radius of gyration (Rg)^77^ and solvent accessible surface area (SASA)^78^ were calculated, considering all heavy atoms of the protein. Distances between residues were calculated using cpptraj, considering the α carbons (Cα) of respective residues.

### Tunnel Analysis

The tunnels were calculated using CAVER 3.0.2^21^ software, which identifies pathways in protein structures by constructing a Voronoi diagram^79^ of their atomic structure. The edges and vertices of such a diagram contain information about the surrounding empty space within the protein. Next, the edges smaller than the user-defined probe radius are removed and the simplified diagram is searched for continuous tunnels from the user-defined starting point to the protein surface using Dijkstra’s algorithm,^80^ with an adjustable cost function that prioritizes shorter and wider ones. When analyzing MD trajectory, tunnels from each frame are clustered based on their similarity to identify tunnel ensembles corresponding to a given pathway.^81^ Here, we have used a 6 Å shell radius and 4 Å shell depth to define the protein surface, specifying the starting point as the center of mass of three catalytic residues (Asn38, Asp109, and His272; numbering corresponds to the crystal structure). A probe radius of 0.9 Å was used to calculate potential tunnels in the enzymes with a time sparsity of 1. Finally, the tunnels were clustered using a clustering threshold of 3.0. Tunnel calculations were performed across 5 μs simulations of both cMD and GaMD, along with their initial fractions, namely, 2.5, 1, and 0.5 μs GaMD simulations for sampling evaluation and comparison between GaMD and cMD methods. Subsequently, TransportTools^82^ (TT) software v0.9.2 was used to generate a unified tunnel network across all variants. Transport tunnels from 5, 2.5, 1, and 0.5 μs GaMD and cMD simulations in the enzyme variants were compared using the comparative analysis module of TT. The clustering method was set to average with a clustering cutoff of 1.

### Reweighting of GaMD Tunnel Profiles

A reweighting protocol was implemented to obtain properties of the investigated tunnels from the original GaMD trajectories, reweighted according to the boost potential applied during simulation. For this purpose, the output files of individual GaMD runs containing the boost potential were parsed along with the corresponding TT profiles in the CSV format for all superclusters, providing access to tunnel characteristics reweighted to the original free energy landscape without bias. The top-100 tunnel conformations, selected based on reweighted TT throughput from each variant, were subjected to further analysis to study the migration of ligands through them with the CaverDock software.^83^

### Cryptic Pocket Detection

To analyze the cryptic and allosteric pockets, three different tools were used. The DeepSite^84^ tool was used to identify viable druggable binding sites on the target protein and pockets likely to bind small molecules. PASSer 2.0^85^ was used to detect probable allosteric site pockets, and FTMove^86^ was used to search for important cryptic binding hotspots utilizing all known conformers of the protein. Web servers for all three tools used the LinB-Wt crystal structure.

### Migration of Ligands Through GaMD Tunnels

To evaluate the efficacy of GaMD tunnels in transporting ligands, CaverDock v1.1 was used to perform molecular docking across the top-100 tunnel conformations with the highest throughput. CaverDock is a computational tool for the study of ligand migrations through protein tunnels by utilizing a modified docking algorithm of AutoDock Vina.^87^ The tunnel is represented by a sequence of spheres that are extracted from CAVER 3.0, and these spheres are then discretized into cross-sectional slices called discs. The ligand is docked at each disc, and its binding energy is calculated using the AutoDock Vina scoring function. This process predicts a migration pathway along the tunnel with an associated estimate of the potential energy.^88^ Here, four ligands, namely bromide ion (Br^–^), 2-bromoethanol (be), 1,2-dibromoethane (dbe), and water (H_2_O), were used to study the transport events in the tunnels, representing the substrate, products, and water important for the catalytic activity of LinB.^16^ MGLTools v1.5.6^89^ was utilized to prepare the inputs using the prepare_receptor4.py and prepare_ligand4.py scripts with default settings. Finally, the continuous upper-bound migration trajectories obtained from CaverDock for each of the top-100 tunnels in each investigated tunnel cluster were considered to calculate the energy barriers for ligand migration and the successful migration of ligands through a particular tunnel cluster using an in-house Python script.^90^

### Distance-Based Principal Component Analysis

Principal component analysis (PCA)^91^ was calculated based on the Cα distances from the side helix (residue IDs: 166–179 in the crystal structure) to the catalytic residue His272 (Figure S1). The Python Scikit-learn package^92^ was used for the calculation of PCA, whereas the distances were calculated using the distance module in cpptraj of AmberTools17. PCA was calculated separately for all three enzyme variants. Cluster analysis was conducted using HDBscan,^92^ with the following parameters: min_cluster_size and min_samples were kept at 60, allow_single_cluster was set to True, and cluster_selection_epsilon was set to 0.5.

## Results

### GaMD Overcomes cMD Sampling Limitations and Enables the Exploration of a More Diverse Conformational Space

To determine the optimal acceleration parameters, short GaMD simulations (50 ns) were tested on LinB-Wt, followed by extended simulations (1000 ns). For LinB-Wt, the total potential boost σ0P was set to 1.3, resulting in a *k*0P of 0.09, which avoided prohibitive instability in the system that was observed with higher σ0P values. Regarding the dihedral potential boost, σ0D was set to 2.5, resulting in a *k*0D of 1.0, which is the maximum possible boost. Consequently, the dual-boost GaMD on LinB-Wt with σ0P = 1.3 and σ0D = 2.5 was performed for all three variants of LinB (Table S1). The relatively low value of *k*0P for our model system LinB could be attributed to its two domains, namely, a more flexible cap domain and a stable core domain. The presence of the flexible cap domain increases the likelihood of multiple tunnels opening in the protein, which can make the enzyme quite sensitive to boost potential energies in GaMD.

**Figure 2:**
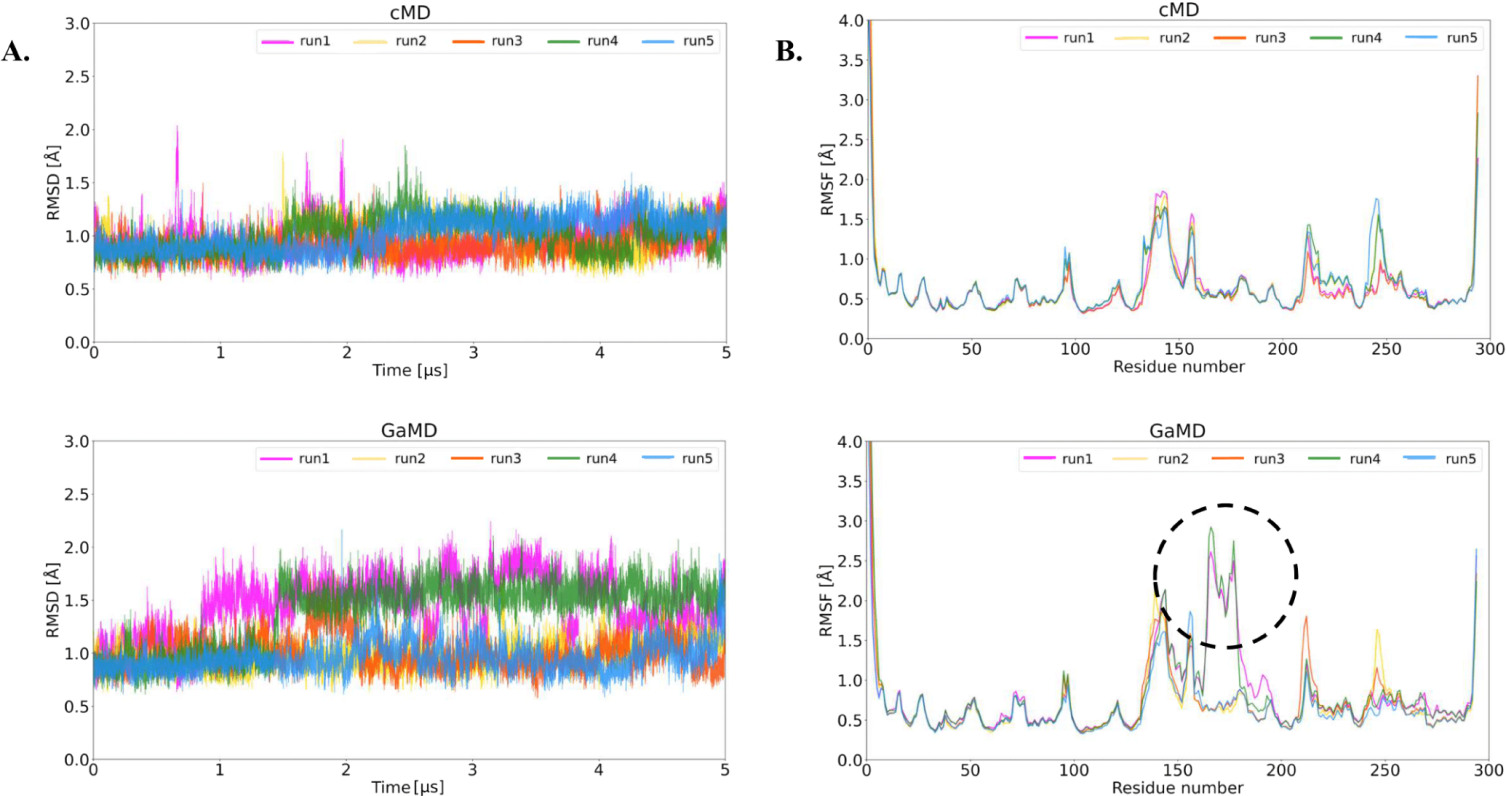
Analysis of protein stability. A. The RMSD time evolution of the LinB-Wt enzyme without the 11-residue long N-terminal tail during cMD and GaMD simulations. B. RMSF of LinB-Wt from cMD and GaMD simulations. The most fluctuating residues from the GaMD simulation are highlighted by a black dashed circle.

To analyze the stability of the enzymes across the simulations, we calculated the RMSD and RMSF for all replicates of the simulations from the investigated LinB variants in both cMD and GaMD simulations. The time evolution of RMSD for the wild-type in cMD and GaMD oscillated below ∼2.5 Å, indicating sufficient equilibration and convergence of the simulations (Figure 2A). Similarly, the LinB mutants indicate even more stable behavior of the systems (Figures S2 and S3) and do not display significant increases in RMSD, suggesting more rigid internal dynamics due to the introduced mutations, even in the cap domain, regardless of the applied boosting potential. Furthermore, to verify the compactness of the protein during simulations, especially upon boosting, we evaluated the Rg and SASA from GaMD trajectories and compared them with cMD, which did not elucidate any significant differences (Figures S4–S6).

After verifying the stability of the protein core, we further evaluated the behavior of the residues forming the catalytic machinery. Therefore, we examined the time-evolution RMSD of individual residues Asn38, Trp109, Asp108, Glu132, and His272 to form the catalytic pentad. Collectively, these residues may have been affected by the additional boost potential in GaMD, thus resulting in the sampling of inactive or unphysical conformations (Figures S7–S11). The most flips are observed in the case of residues Asp108, Glu132, and His272. Specifically, for His272 and Glu132, the His272 backbone forms a hydrogen bond with Glu132. However, during the simulation, this hydrogen bond breaks, leading to these residues adopting different conformational states before returning to the initial conformation or, sometimes, the His272 ring entering a different conformational state, causing fluctuations in the RMSD plot (Figures S7–S11). In the case of Asp108, the side chain frequently fluctuates between different conformations, with an RMSD range of approximately 1.25 to 1.50 Å. When considered separately and cumulatively in both cMD and GaMD, the values range between 2.0 and 2.5 Å, indicating that the enzymes’ catalytic machineries are not distorted due to the added boost potential in GaMD.

### GaMD Enabled the Accurate Detection of the Tunnel Network Known for LinB and Led to the Discovery of a New Side Tunnel (ST)

**Figure 3:**
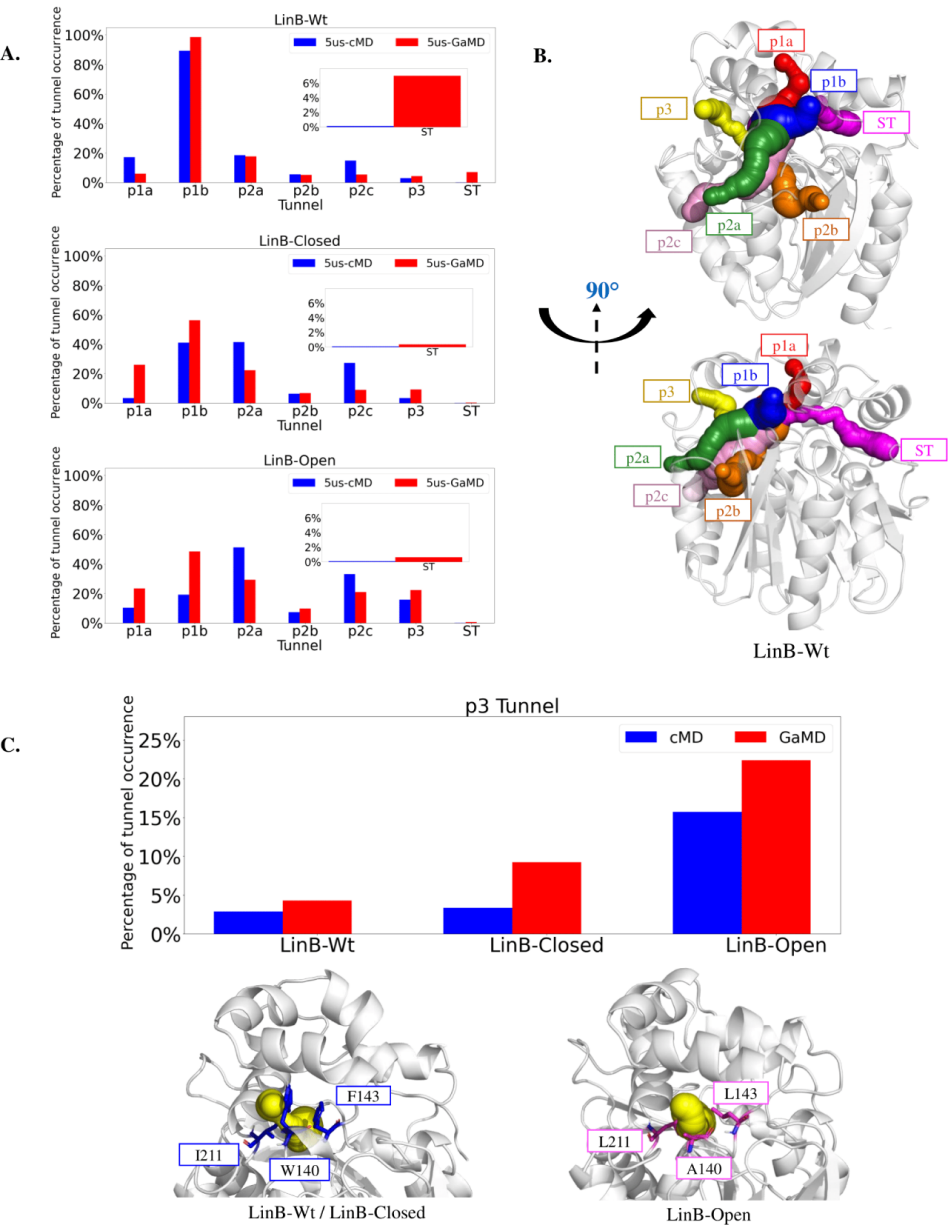
Occurrence of tunnels in LinB variants. A. Percentage of tunnels being open in the ensemble for LinB-Wt, LinB-Closed, and LinB-Open variants. Inset plots present the increased sampling of ST captured by GaMD in LinB-Wt. B. Representation of the LinB-Wt crystal structure (gray cartoon) and the tunnel network in the LinB family (colored spheres). The shown representative tunnels were selected from GaMD simulations of LinB-Wt to have their bottleneck radius and length matching the average properties of individual tunnel clusters observed in these simulations. C. Plot of the p3 tunnel from TT analysis in respective Wt and mutants with cMD and GaMD methods (top) and representation of the protein structure region where the p3 tunnel opens in Wt and LinB-Closed mutants, containing bulky residues TRP, PHE, and ILE; also, the region of p3 tunnel opening in LinB-Open, containing the mutation W140A+F143L+I211L, which leads to the widening of this region (bottom). The representatives of p3 tunnels were selected from GaMD simulations of LinB-Wt and LinB-Open, respectively, to have their bottleneck radius and length match the average properties of their tunnel clusters observed in these simulations.

We calculated the tunnels to investigate the internal dynamics of ligand transport pathways due to enhanced sampling. The tunnel networks were detected using a combined approach, including CAVER 3.0.2 calculations and further tunnel unification across all simulated systems and replicates in TransportTools. This analysis revealed the presence of all known branches of the p1 and p2 tunnels, namely, p1a, p1b, p2a, p2b, and p2c, respectively. Additionally, two rare tunnels – known as p3 and the newly discovered side tunnel (ST) – were found (Figure 3A,B). The newly discovered ST opening corresponds to a region with high RMSD fluctuations, displaying increased values in two replicates of GaMD (especially replicates 1 and 4), due to the boosting potential helping the enzyme to explore a broader sampling space. This observation is consistent with the RMSF profiles showing increased fluctuations in the cap region (residue numbers 166–179) for these corresponding replicates, suggesting improved sampling (Figure 2B). Furthermore, to compare the sampling efficiency between cMD and GaMD during the time evolution of the simulation, we considered various fractions of the trajectories, including the full length (5 μs), first half (2.5 μs), initial 20% (1 μs), and 10% (500 ns). We noted that at least 1 μs of GaMD is required to observe enhanced exploration of tunnels, which was particularly noticeable for the transient tunnel ST (Figures S12–S14). GaMD enhances exploration of the sampling space to capture the rare tunnels more effectively than cMD (Figure 3A). Besides the tunnel occurrence as defined by the probe radius used for the CAVER calculations (0.9 Å), we also considered other, mostly geometric, properties of the tunnels, such as average length, average bottleneck radius, and maximum bottleneck radius. Interestingly, although tunnels exhibit consistent geometric properties within various groups, it was observed that GaMD simulations tend to open tunnels more, in particular, the primary p1b tunnel (Figures S15–S17), resulting in an increased maximum bottleneck radius exceeding 3 Å (Figures S12–S14).

The results obtained from GaMD simulations were compared with the tunnel network in three variants with already published data on de novo tunnel engineering.^16^ We found that the p3 tunnel opening follows a trend similar to that in previous studies; that is, the characteristic tunnel network known for each variant is reproduced in the GaMD trajectories. In LinB-Wt, the p1 tunnel branches serve as the primary conduits, which undergo changes in mutants due to the closure of p1 tunnels, resulting in a significant decrease in their occurrence. Additionally, we observed a significant increase in the presence of the p3 tunnel in the LinB-Open variant due to p3-opening mutations. Interestingly, the GaMD tunnels have a tendency for more frequent and broader tunnel openings (Figure 3C), particularly in the case of the p3 tunnel (as indicated by the high bottleneck radius shown in Figures S12–S17). Furthermore, we observed increased sampling of the ST in the wild-type protein, with its occurrence being comparable to the rare p3 tunnel in GaMD simulations (Figure 3A), which was not as pronounced in mutants. Moreover, although it is sampled by cMD, we noted its increased sampling, mostly in GaMD for LinB-Wt, whereas the other two variants do not show such prominent ST openings (Figure 3A). Due to the large structural perturbation in the helix caused by boosted GaMD simulation, the opening of a transient ST is enhanced. We monitored the RMSF of the protein throughout the simulation and found an increased fluctuation in the cap domain in two out of five GaMD simulation replicas, indicating that GaMD visited a broader conformational space.

### Insights into the Functional Relevance of the Discovered Side Tunnel (ST)

**Figure 4:**
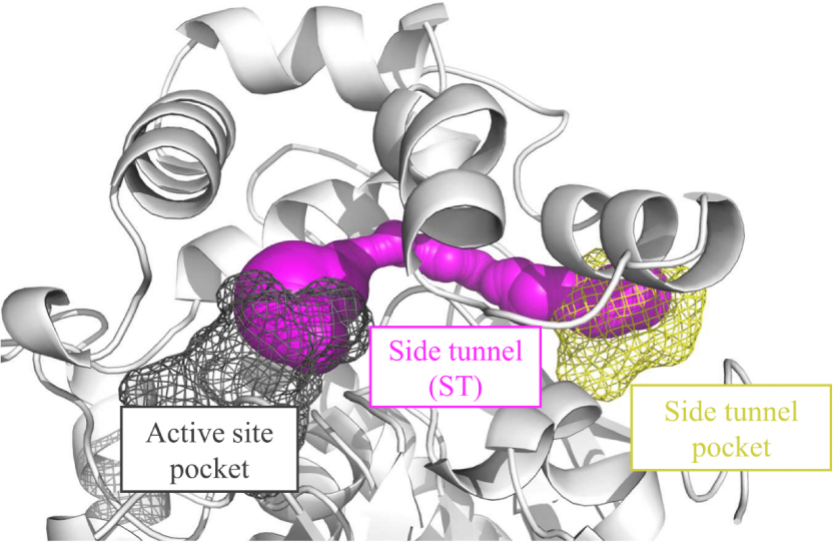
Cryptic pockets detected in the LinB-Wt structure by FTMove. The active site and ST pockets are shown in dark gray and yellow, respectively. The ST pocket is located at the mouth of the ST, which connects these two pockets. The shown representative ST was selected from GaMD simulations of LinB-Wt to have its bottleneck radius and length match the average properties of its tunnel clusters observed in these simulations.

In addition to tunnels, the ligand interaction with enzyme surface pockets or cryptic pockets has been considered important because pocket residues can influence ligand transport through tunnels.^93^ Interestingly, Raczyńska et al.^93^ published a study focused on identifying transient binding sites on the enzyme surface as potential sites for engineering enzyme activity. Their study, conducted on LinB-Wt, indicated the ability of the ST entrance pocket to bind 1-chlorohexane. Furthermore, they showed that the experimentally tested mutation of the ST entrance pocket residue A189F increased the enzyme activity by 21.4% for 1-chlorohexane and 26.2% for 1-bromocyclohexane,^93^ which highlights the importance of the ST pocket and the newly discovered ST path, which connects the active site or active site pocket to the ST pocket for LinB (Figure 4). To study the opening site of the ST at the enzyme surface and its connectivity with the active site, we used cryptic and allosteric pocket detection tools, such as FTMove, PASSer 2.0, and DeepSite. Our analysis revealed a cryptic pocket at the mouth of the ST as one of the major ligand-binding cryptic pockets, suggesting potential allosteric communication via the ST pocket. DeepSite predictions of cryptic pockets showed the ST pocket with the second-highest score after the active site p1 tunnel pocket (Figure S18). Similar results were obtained using the PASSer 2.0 allosteric site prediction tool (Figure S19) and FTMove, where the p1 tunnel pocket was ranked third, the active site pocket fourth, and the ST pocket second among all of the different cryptic pockets detected by the tool (Figure S20).

### ST Can Transport Ligands Similarly to the P3 Tunnel

**Figure 5:**
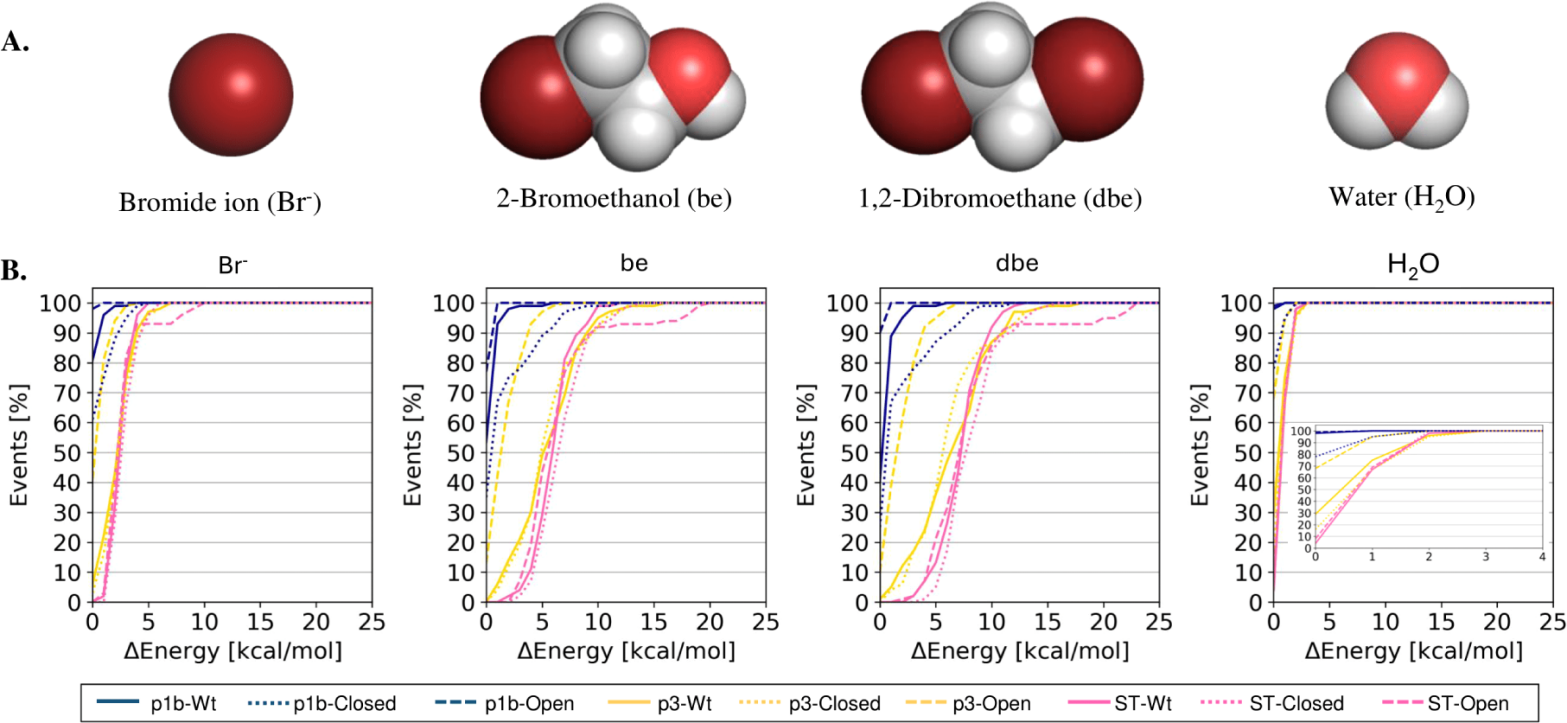
Energetic analysis of ligand transport through the transient ST. A. The structures of the four ligands studied: 1,2-dibromoethane (dbe), 2-bromoethanol (be), bromide ion (Br^–^), and water (H_2_O), used for transport analysis (top). B. Plots showing the energy costs of successful transport events in all three variants, LinB-Wt, LinB-Open, and LinB-Closed. Blue represents transport events across the top-100 conformations of p1b tunnels, yellow represents transport events across the top-100 conformations of p3 tunnels, and pink shows transport events across the top-100 conformations of the ST (bottom).

We observed that the ST is connected to a cryptic pocket, the functional relevance of which has been experimentally verified by mutagenesis.^93^ Hence, we were interested in probing to what extent the ST transport viability for four relevant substrate and product molecules (Figure 5A) matches those of the auxiliary p3 and primary p1b tunnels. As the explicit simulations of transport via gated tunnels of LinB variants were shown to be very hard to execute even for its primary p1b tunnel and a single product molecule,^43^ performing rigorous simulations with four molecules and three tunnels, where the ST opening requires rather pronounced protein conformational change, would represent an extremely complex and computationally intensive task. Instead, we opted for estimating the relative transport propensity of these tunnels with CaverDock, which was successfully applied for predicting ligand unbinding rates,^83,94^ estimating effects of mutations on ligand transport,^83,95,96^ and approximating energy profiles in agreement with insights from sophisticated enhanced sampling simulations.^96^

Here, we performed these docking calculations using ensembles of the top-100 conformations of tunnels with the highest throughput with each of the four investigated molecules to compare the efficiency of the transient ST in transporting these molecules with the p3 and p1b tunnels. The top-100 tunnel conformations used for calculations were obtained from the GaMD trajectories, and using CaverDock, we calculated the energy profile by assessing the binding energy of the ligand to the protein along the entire tunnel length. Next, we calculated the energy barriers that the ligands must overcome for these 100 considered transport events (Supporting Information File 2). This energetic evaluation of transport demonstrated that the ST was able to transport all four ligands, and interestingly, the energetics of these transports was comparable to the energy barriers sampled for the auxiliary tunnel p3 (Figure 5B). Our evaluation indicated the importance of the ST in conjunction with the known p3 tunnel. Similar observations were obtained for haloalkane dehalogenase DhaA, where an equivalent ST was also repeatedly identified in energy-unbiased high-throughput MD simulations.^97^ This tunnel showed capacity for transporting water molecules at levels comparable to that of p3.^97^ Additionally, the energy barrier for water transport (H_2_O) was found to be lower (<5 kcal/mol) in all three tunnels, which shows that all the tunnels were effectively able to transport water through the enzyme. Furthermore, the best-performing tunnel was identified as p1b from the Open mutant, consistent with studies on de novo tunnel engineering.^16^

### ST Opening Mechanism is Different in LinB-Wt and Its Mutants

**Figure 6:**
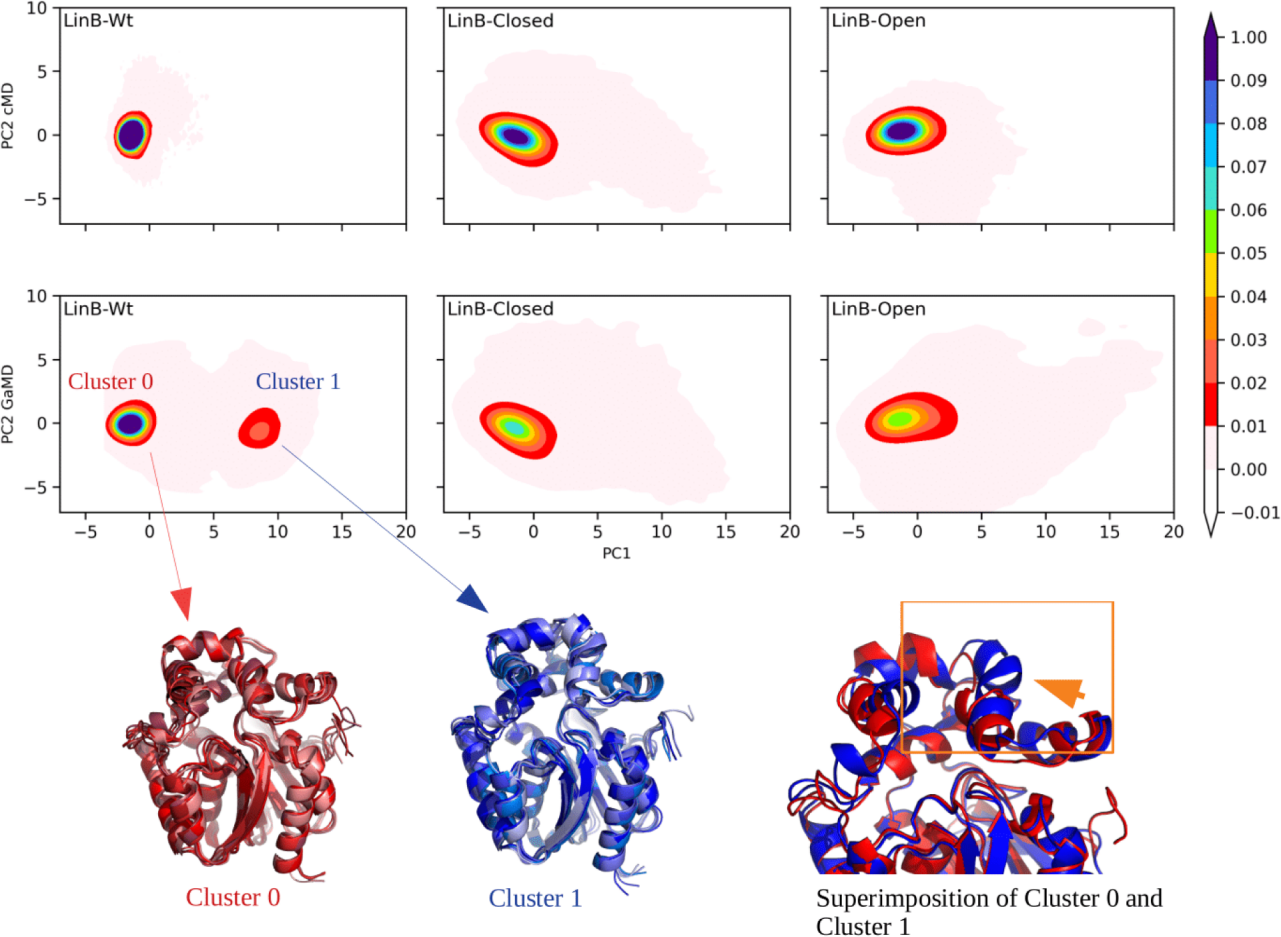
Detection of distinct conformational states sampled by LinB variants. Distance-based PCA: PC1 and PC2 were plotted for all three variants of LinB and represented as Clusters 0 and 1 (all representative frames) from the LinB-Wt GaMD simulation, showing two different states of the protein. In the superimposed structures, red represents Cluster 0, and blue represents Cluster 1, showing the different conformational states captured by LinB-Wt.

The importance and validity of the ST are confirmed by our results. Therefore, to understand the conformational changes associated with ST opening, we conducted a distance-based PCA in LinB-Wt and its mutants, focusing on the most dynamic region of the protein as confirmed through RMSF, as this approach was shown to be well suited for the investigation of functionally relevant conformational changes.^98−100^ In all cases, the first two principal components (PCs) explain at least 80% variance in the original data (Figure S21), suggesting that they were sufficient for further analyses. In LinB-Wt, we found that GaMD simulations sample two distinct conformational states, in contrast to cMD simulations (Figure 6), indicating that this side helix region provides the protein with the possibility to visit different conformational states (Figure S22). In cMD simulations, we also detected the ST occurrence, but it was less frequent, which confirms that the opening is not forced by the biasing potential but highlights that the system requires broad sampling to capture it efficiently. Major conformational states in all variants were further investigated by HDBscan clustering of distance-based PCA data. We found two predominant states for LinB-Wt in GaMD (Figure 6), which were also partially observed for mutants but only sporadically (Figures S23–S25). Importantly, this analysis further highlights the improved sampling in GaMD trajectories compared to that of cMD.

**Figure 7:**
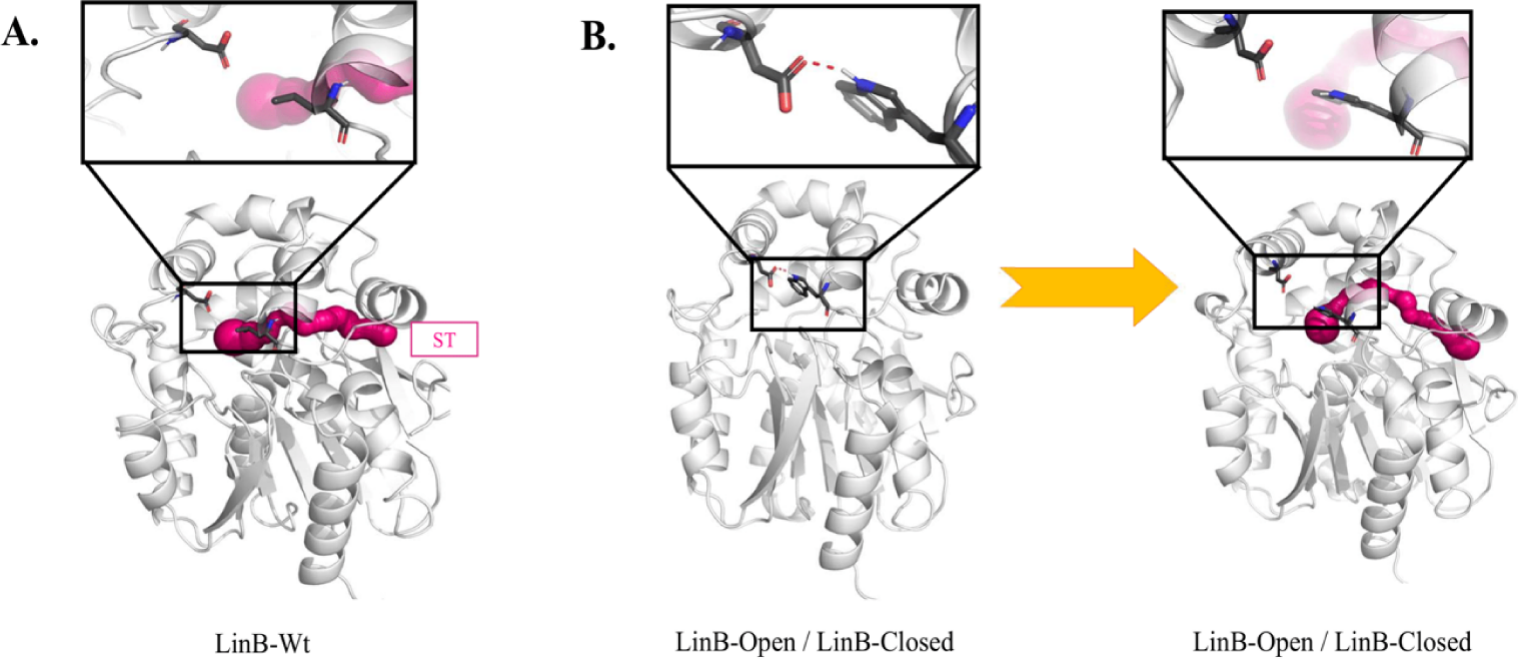
Mechanism of side tunnel opening. A. Representation of the ST in the LinB-Wt protein showing the necessary movement of the side helix for the opening of the ST. B. Representation of the ST in mutants showing the necessary movement of the side helix accompanied by the breakage of the hydrogen bond between Trp176 and Asp146.

We investigated in-depth the mechanism of the rare ST opening in LinB-Wt and mutants. In LinB-Wt, the prerequisite for the opening of the ST is the movement of the side helix away from the protein cap domain. Due to the high fluctuation of the side helix of the cap domain, the ST opens in LinB-Wt frequently (Figures 7A and S26), especially in GaMD-boosted simulations. In the case of mutants (LinB-Closed and LinB-Open), a mutation at the bottleneck residue of the ST (L177W) introduces hydrogen bonding with D147, and the breakage of this hydrogen bond promotes the opening of the ST. However, this process is notably more energetically unfavorable, resulting in the ST being less frequently open in mutants compared to the wild-type enzyme (Figures 7B, S27, and S28). We had already observed that LinB-Wt samples the ST more frequently than mutants because, in the case of LinB-Wt, the lack of a hydrogen bond donor makes the movement of the side helix away from the protein easier, contributing to the opening of the side tunnel. In contrast, in mutants due to hydrogen bonding, the opening is less favorable and, therefore, less frequent.

## Discussion

In this study, we discussed the potential application of the GaMD enhanced sampling method to investigate tunnels and ligand transport pathways in LinB dehalogenases. Our findings demonstrate that GaMD is not only able to identify all known tunnels (p1a, p1b, p2a, p2b, and p2c) comparable to the cMD method but also enhances sampling of the enzyme conformational space and visits rare tunnels (ST and p3 tunnel). The improved sampling provided by GaMD enabled us to identify a previously unexplored ST and to further understand the mechanism of its opening in the three LinB variants. This opening mechanism was found to be clear and straightforward in the case of the LinB-Wt enzyme but more complex in mutants. Importantly, such a thorough investigation of the newly discovered ST pathway would not have been possible without the sufficient conformational exploration provided by GaMD. What is critically important is that this method not only provided a better picture of conformational dynamics for the three LinB variants but also facilitated consistent exploration of known tunnels from broad computational and experimental data for all variants.

Our exploration revealed that in LinB-Wt, the movement of the side helix is the main contributing factor to the opening of the ST pathway. For mutants, the sampling of ST pathways is less pronounced because of the L177W mutation, resulting in a hydrogen bond between introduced W177 and D147. This prohibits the opening of the helix observed in the wild-type enzyme and proves that the ST is mostly unfavorable in mutants. Thus, it is rarely observed in standard and enhanced MD simulations. Furthermore, the functional importance of this ST pathway is supported by the exploration of druggable, cryptic, and allosteric pockets, all consistently pointing to the mouth of the ST pathway as the functional site. This is further corroborated by a recent experimental study demonstrating the ability of the ST pathway mouth to bind drugs.^93^ Additionally, migration analysis using CaverDock with GaMD tunnels confirmed the capacity of the transient ST to transport the substrate and product molecules efficiently at levels comparable to those of auxiliary tunnel p3, which supports the auxiliary role of the ST in LinB.

Regarding the p3 tunnel, we observed that GaMD provides better sampling compared with standard MD simulations. Furthermore, we noted the trend expected for LinB-Wt and mutant enzymes from previous literature data. The p3 tunnel is detected more frequently in the LinB-Open mutant due to the mutations W140A, F143L, and I211L, which replace bulky residues with smaller ones, aiming to provide more space for the opening of the p3 tunnel. Conversely, in LinB-Wt and the LinB-Closed mutant, the amino acids at the mouth of the p3 tunnel are not modified, resulting in a more restricted opening compared with the LinB-Open variant.

## Conclusions

Our study demonstrates GaMD as a practically useful approach for investigating tunnels in proteins. By overcoming the sampling limitations of standard MD simulations, GaMD can more effectively explore the conformational dynamics of the protein under study while limiting computational costs. This opens up new possibilities for identifying tunnels by efficiently sampling rare events and overcoming unfavorable energy barriers. Therefore, as shown for the LinB enzyme in our study, this method is suitable for effectively exploring new pathways that can become a druggable site to target in addition to the conventional targeting of the active site itself.

## Data Availability

Underlying data are available on the Zenodo repository: (i) https://zenodo.org/doi/10.5281/zenodo.11092891, containing input, output, and analysis files; (ii) https://zenodo.org/doi/10.5281/zenodo.11093856, containing the parameter files and stripped MD trajectory files.

## Supporting Information Available

Testing parameters for GaMD simulations (Table S1); the region used for distance-based PCA calculation (Figure S1); RMSD and RMSF of mutant enzymes (Figures S2 and S3); Rg and SASA of LinB-Wt and its mutants (Figures S4–S6); RMSD of catalytic residues (Figures S7–S11); comparison between cMD and GaMD tunnel properties from TransportTools (Figures S12–S14); Bottleneck radius distribution analysis (Figures S15–S17); the results of cryptic and allosteric pocket detecting tools (Figures S18–S20); variance and cumulative variance of all fourteen principal components in cMD and GaMD (Figure S21); plots showing PCA comparison between cMD and GaMD (Figure S22); HDBscan cluster analysis of LinB-Wt and its mutants (Figures S23–S25); mechanism of side tunnel opening using helix movement in LinB-Wt (Figure S26); mechanism of side tunnel opening using helix movement and hydrogen bond analysis in the mutant enzymes (Figures S27 and S28) (PDF)
Upper bound energy profiles using CaverDock from individual 100 tunnels in all variants with all four ligands (PDF)

## Abbreviations

MD: molecular dynamics
cMD: conventional MD
GaMD: Gaussian accelerated MD
RMSD: root mean square deviation
RMSF: root mean square fluctuation
TT: TransportTools
ST: side tunnel
EC: Enzyme Commission
ABF: adaptive biasing force
CV: collective variable
NMR: nuclear magnetic resonance
aMD: accelerated MD
PC1: principal component 1
PC2: principal component 2
cryo-EM: cryogenic electron microscopy
RAMD: random accelerated MD
LiGaMD: ligand Gaussian accelerated MD
