# Supplementary material for: Reinforcing Tunnel Network Exploration in Proteins Using Gaussian Accelerated Molecular Dynamics
Source: J Chem Inf Model. 2024 Aug 15;64(16):6623–35. doi: 10.1021/acs.jcim.4c00966 (PMC11351021; doi:10.1021/acs.jcim.4c00966)

Supporting Information File 2

Reinforcing Tunnel Network Exploration in Proteins Using Gaussian Accelerated Molecular Dynamics

Nishita Mandal[1,2], Bartlomiej Surpeta[*1,2] and Jan Brezovsky[*1,2]

1. Laboratory of Biomolecular Interactions and Transport, Department of Gene Expression, Institute of Molecular Biology and Biotechnology, Faculty of Biology, Adam Mickiewicz University, Uniwersytetu Poznanskiego 6, 61-614 Poznan, Poland
2. International Institute of Molecular and Cell Biology in Warsaw, Ks Trojdena 4, 02-109 Warsaw, Poland

Corresponding authors

Bartlomiej Surpeta, E-mail: bartlomiej.surpeta@amu.edu.pl

Jan Brezovsky, E-mail: janbre@amu.edu.pl

KEYWORDS

transport tunnel, molecular dynamics, haloalkane dehalogenase, enhanced sampling methods, mechanism

Caverdock energy profiles of individual 100 tunnels in all variants.

1. Upper Bound energy profile of p1b-Wt with Bromide ion (Br^-) ligand. The X-axis represents upper bound energy (kcal/mol) and Y-axis represents length of the trajectory [Å], along the disc of tunnel.

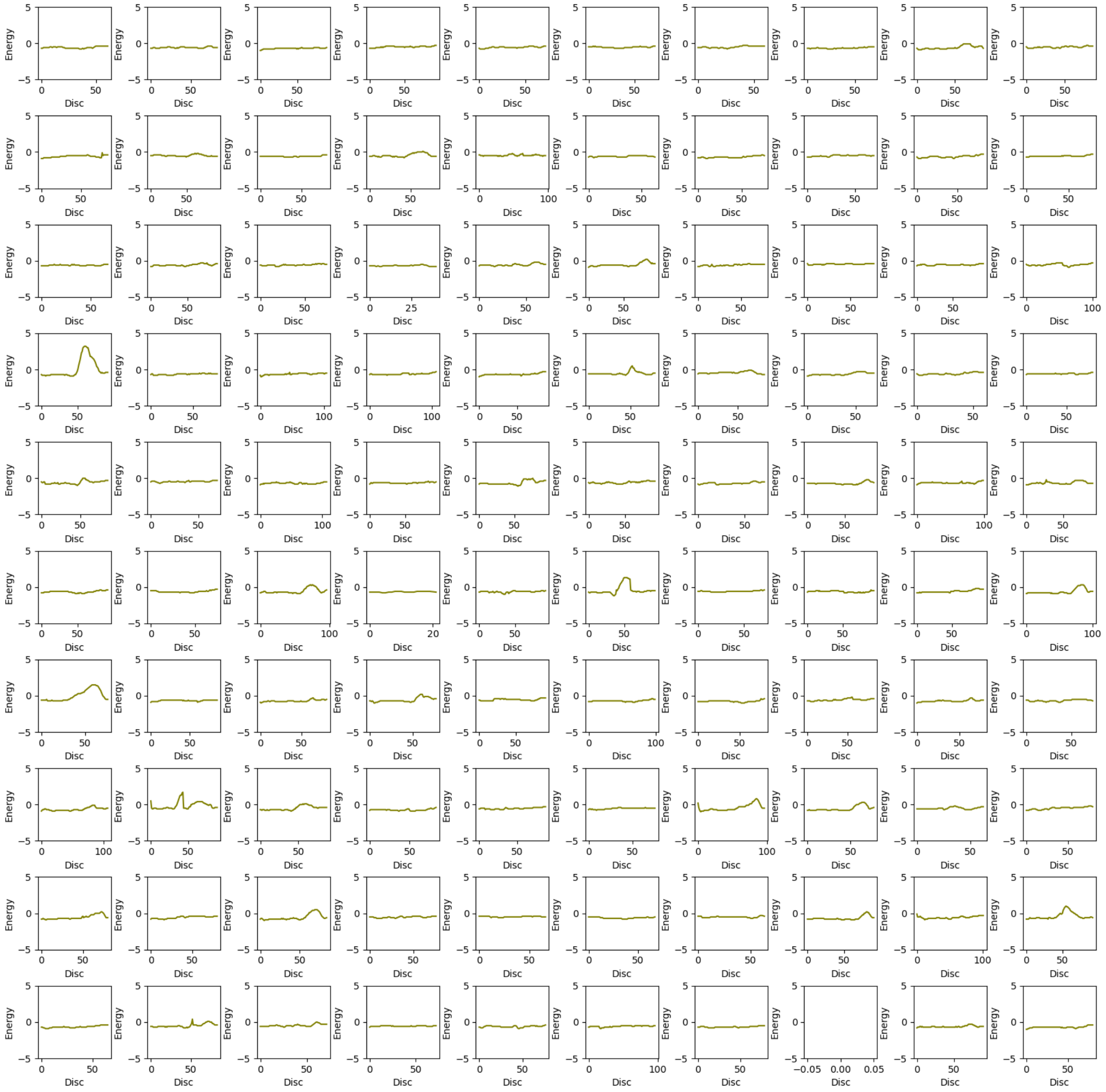

2. Upper Bound energy profile of p1b-Wt with 2-Bromoethanol (be) ligand. The X-axis represents upper bound energy (kcal/mol) and Y-axis represents length of the trajectory [Å], along the disc of tunnel.

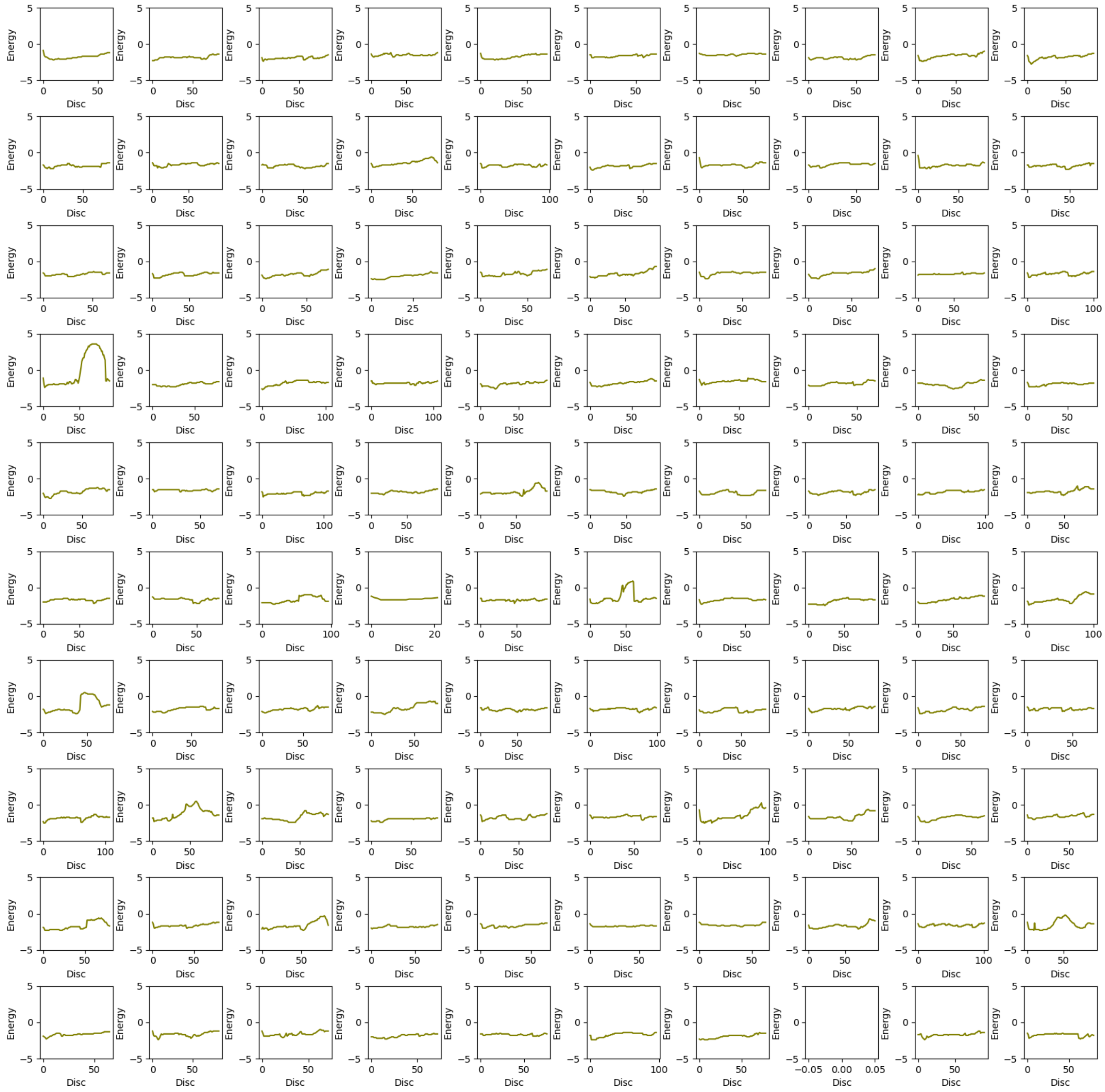

3. Upper Bound energy profile of p1b-Wt with 1,2- Dibromoethane (dbe) ligand. The X-axis represents upper bound energy (kcal/mol) and Y-axis represents length of the trajectory [Å], along the disc of tunnel.

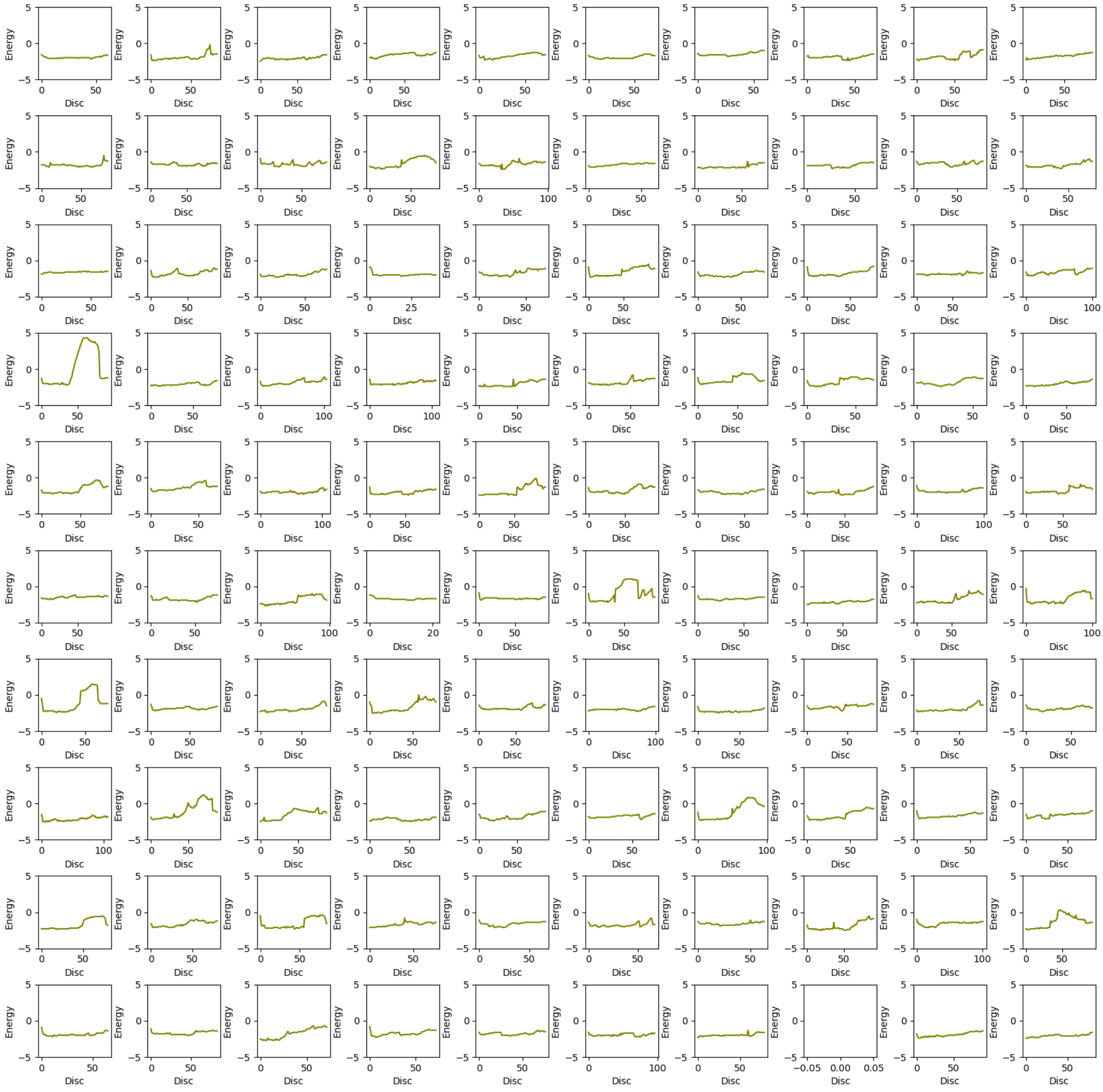

4. Upper Bound energy profile of p1b-Wt with Water (H_2O) ligand. The X-axis represents upper bound energy (kcal/mol) and Y-axis represents length of the trajectory [Å], along the disc of tunnel.

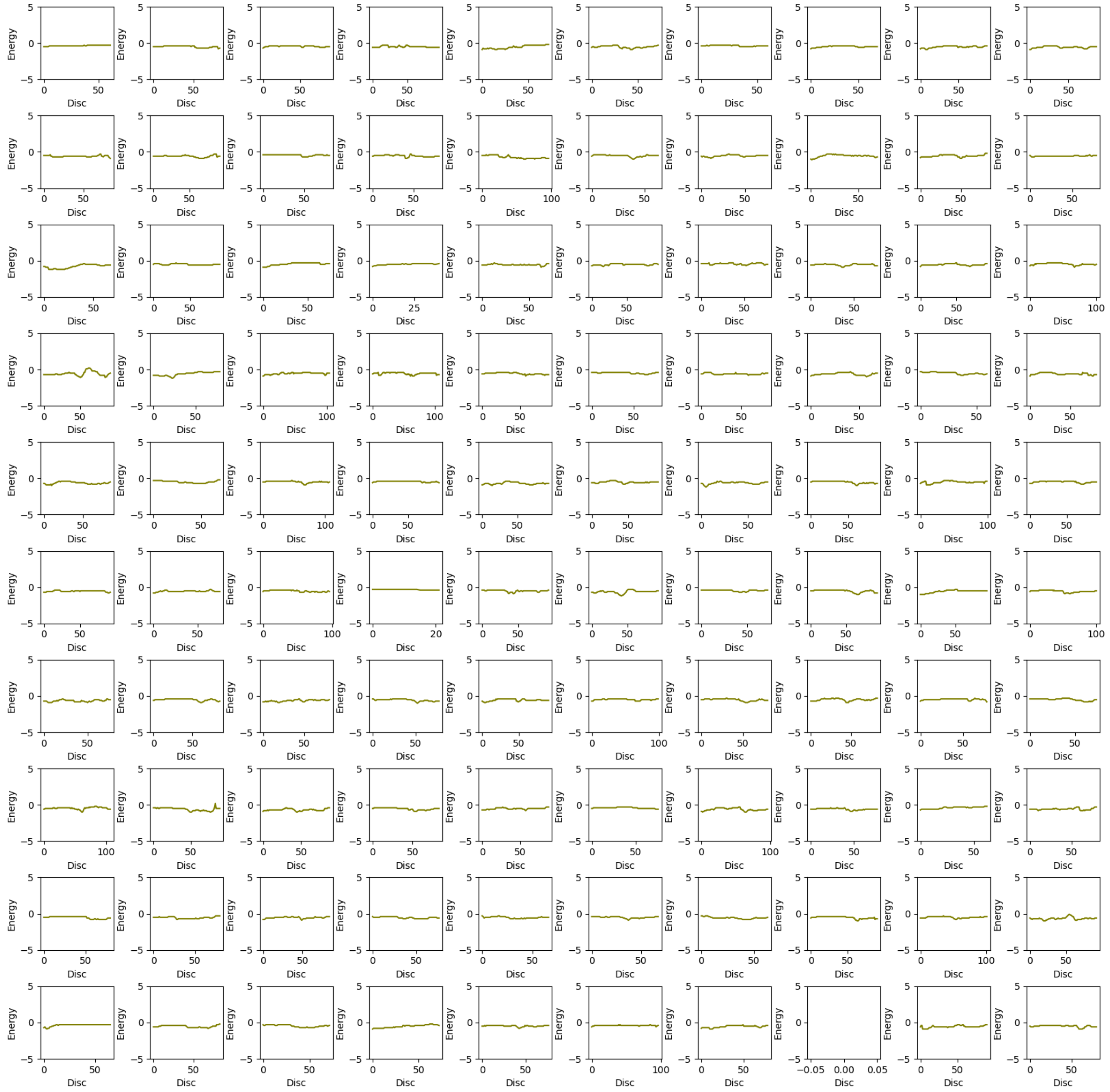

5. Upper Bound energy profile of p1b-Closed with Bromide ion (Br^-) ligand. The X-axis represents upper bound energy (kcal/mol) and Y-axis represents length of the trajectory [Å], along the disc of tunnel.

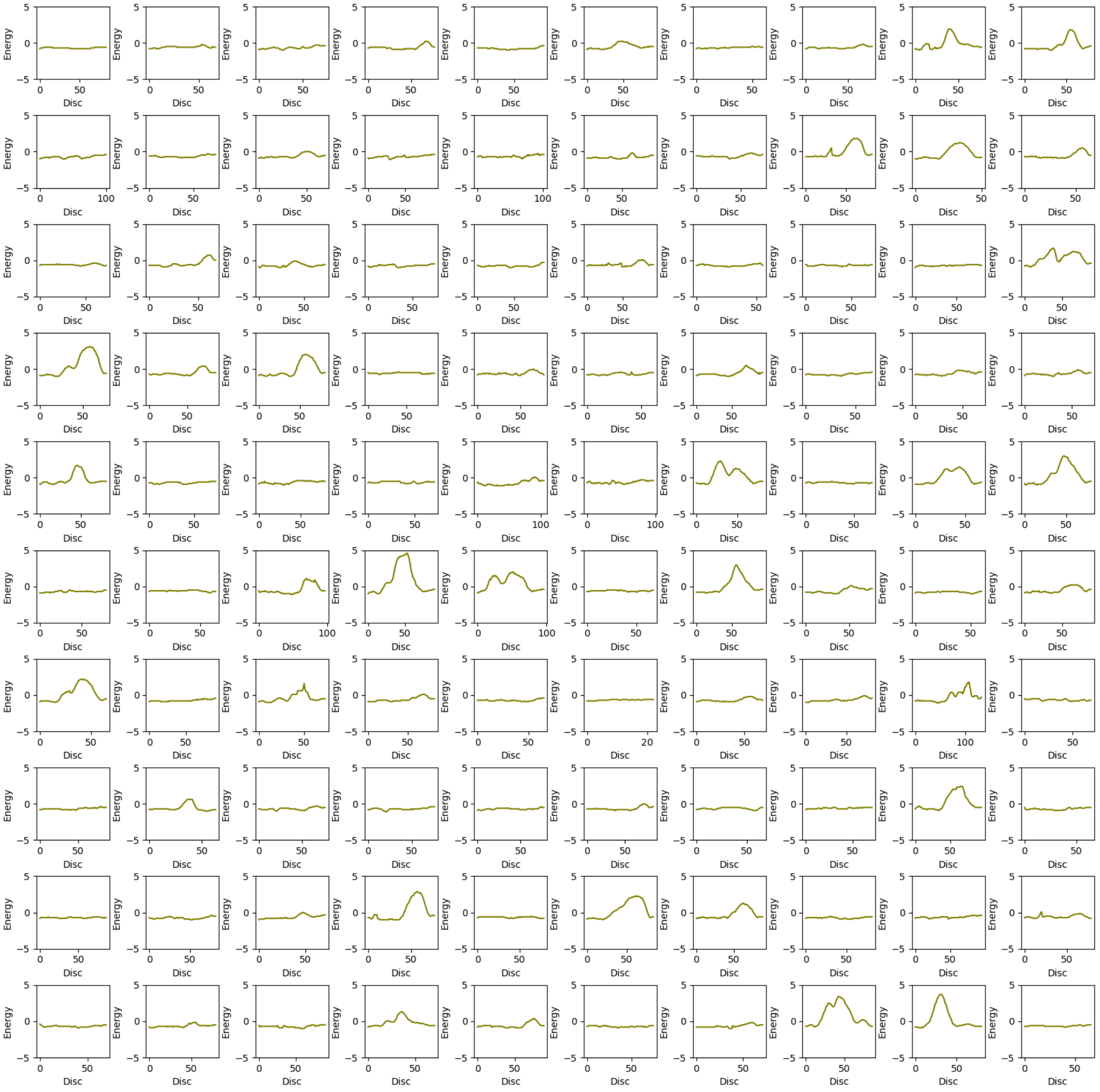

6. Upper Bound energy profile of p1b-Closed with 2-Bromoethanol (be) ligand. The X-axis represents upper bound energy (kcal/mol) and Y-axis represents length of the trajectory [Å], along the disc of tunnel.

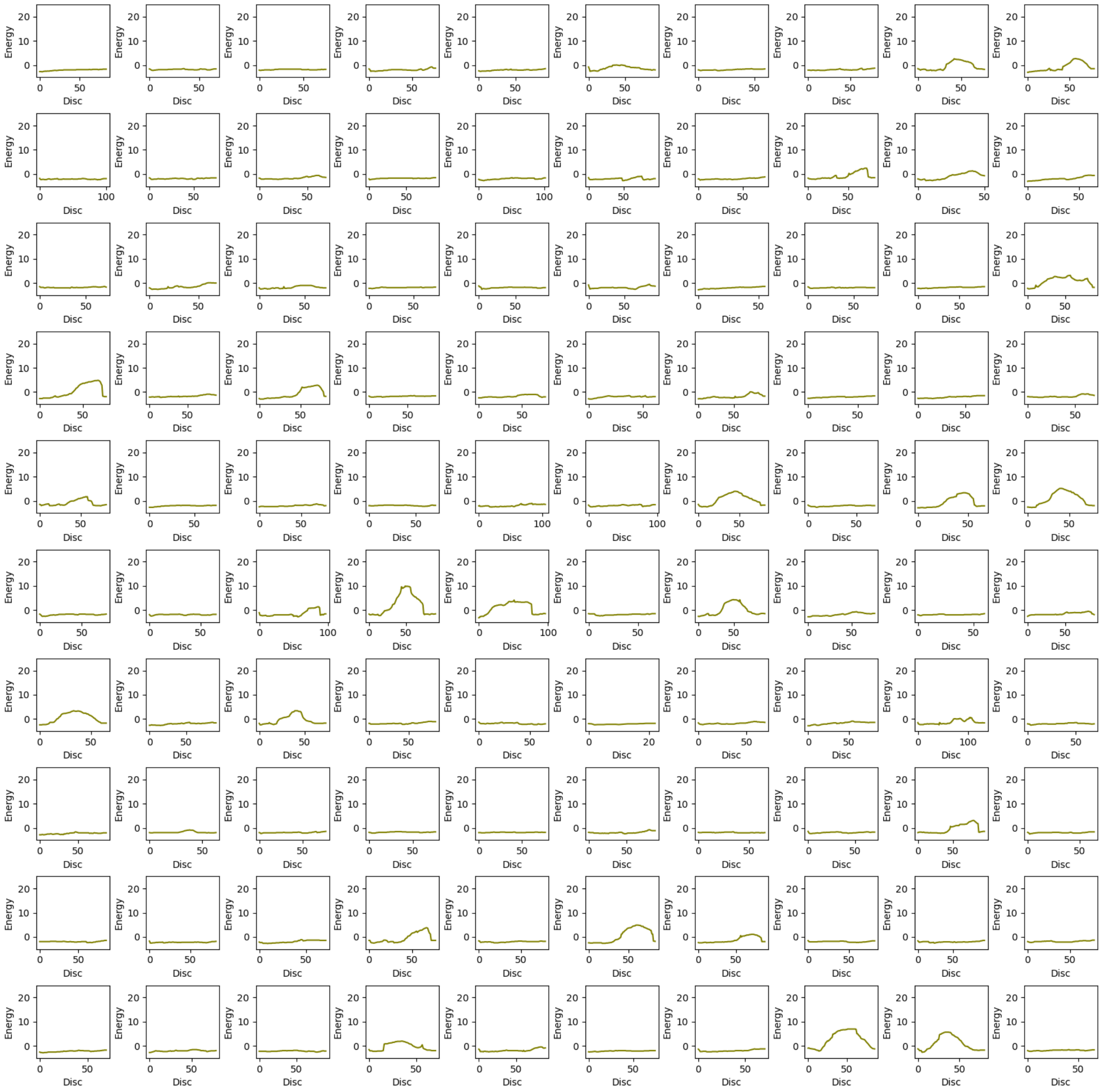

7. Upper Bound energy profile of p1b-Closed with 1,2- Dibromoethane (dbe) ligand. The X-axis represents upper bound energy (kcal/mol) and Y-axis represents length of the trajectory [Å], along the disc of tunnel.

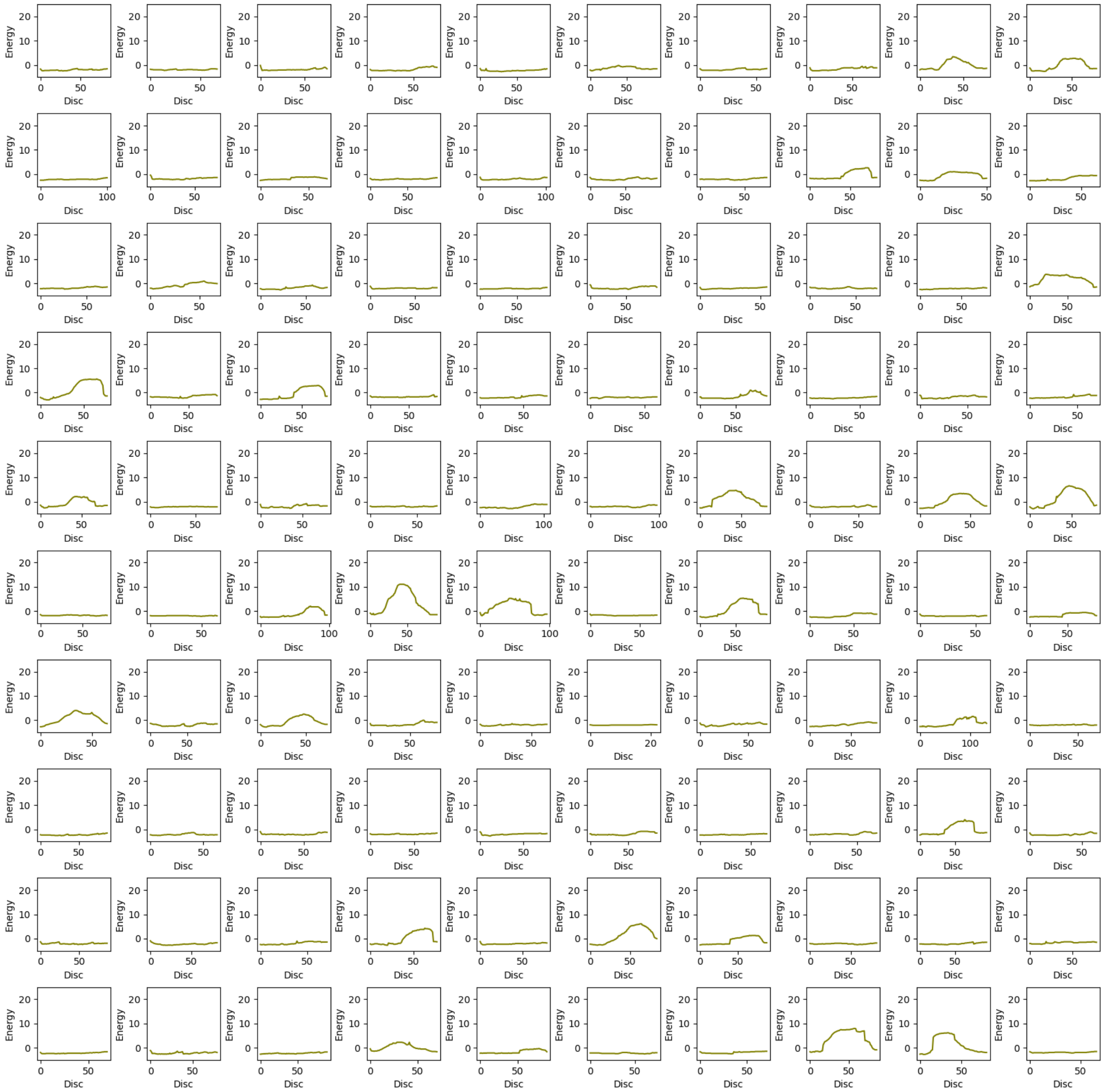

8. Upper Bound energy profile of p1b-Closed with Water (H_2O) ligand. The X-axis represents upper bound energy (kcal/mol) and Y-axis represents length of the trajectory [Å], along the disc of tunnel.

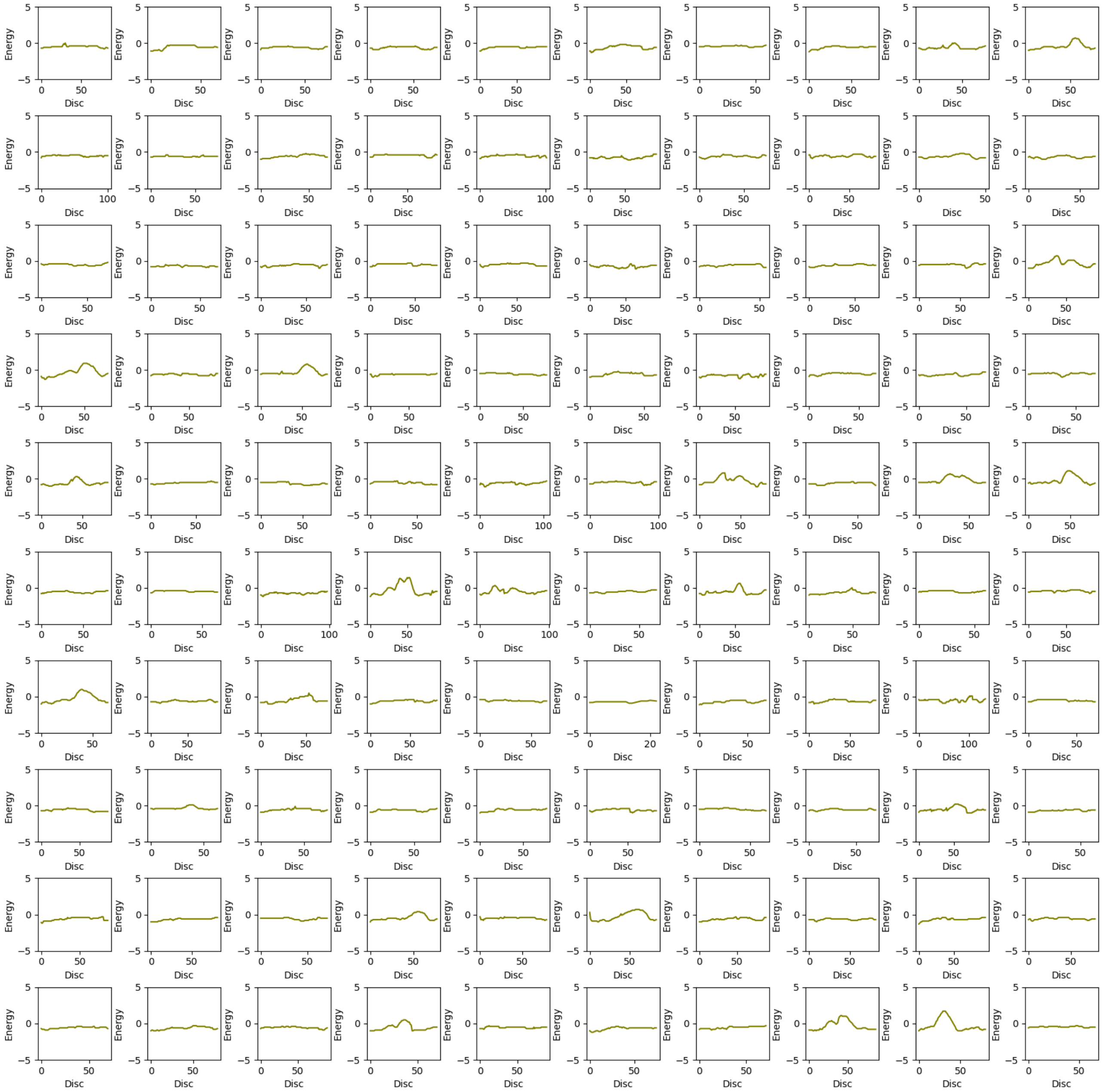

9. Upper Bound energy profile of p1b-Open with Bromide ion (Br^-) ligand. The X-axis represents upper bound energy (kcal/mol) and Y-axis represents length of the trajectory [Å], along the disc of tunnel.

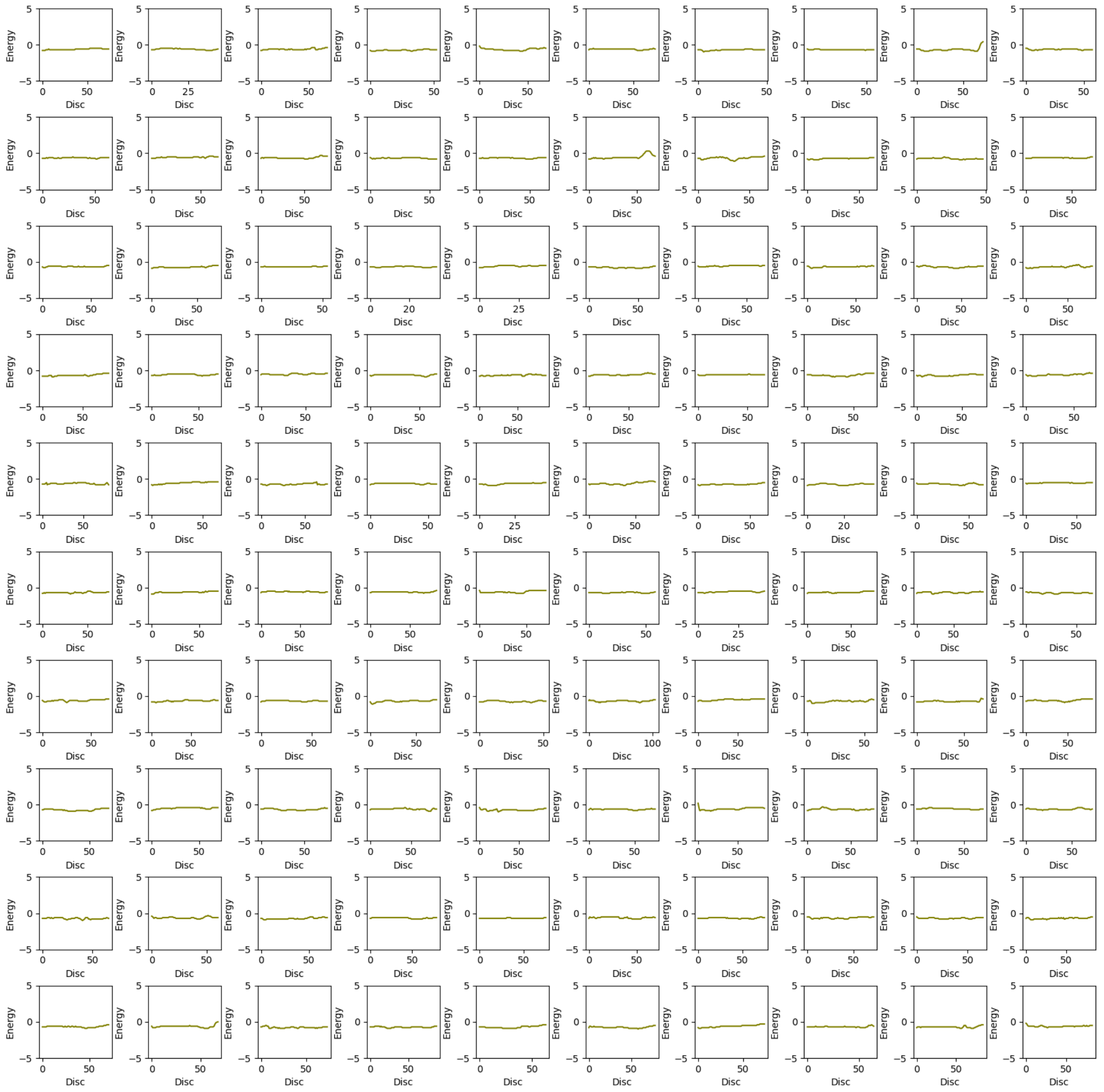

10. Upper Bound energy profile of p1b-Open with 2-Bromoethanol (be) ligand. The X-axis represents upper bound energy (kcal/mol) and Y-axis represents length of the trajectory [Å], along the disc of tunnel.

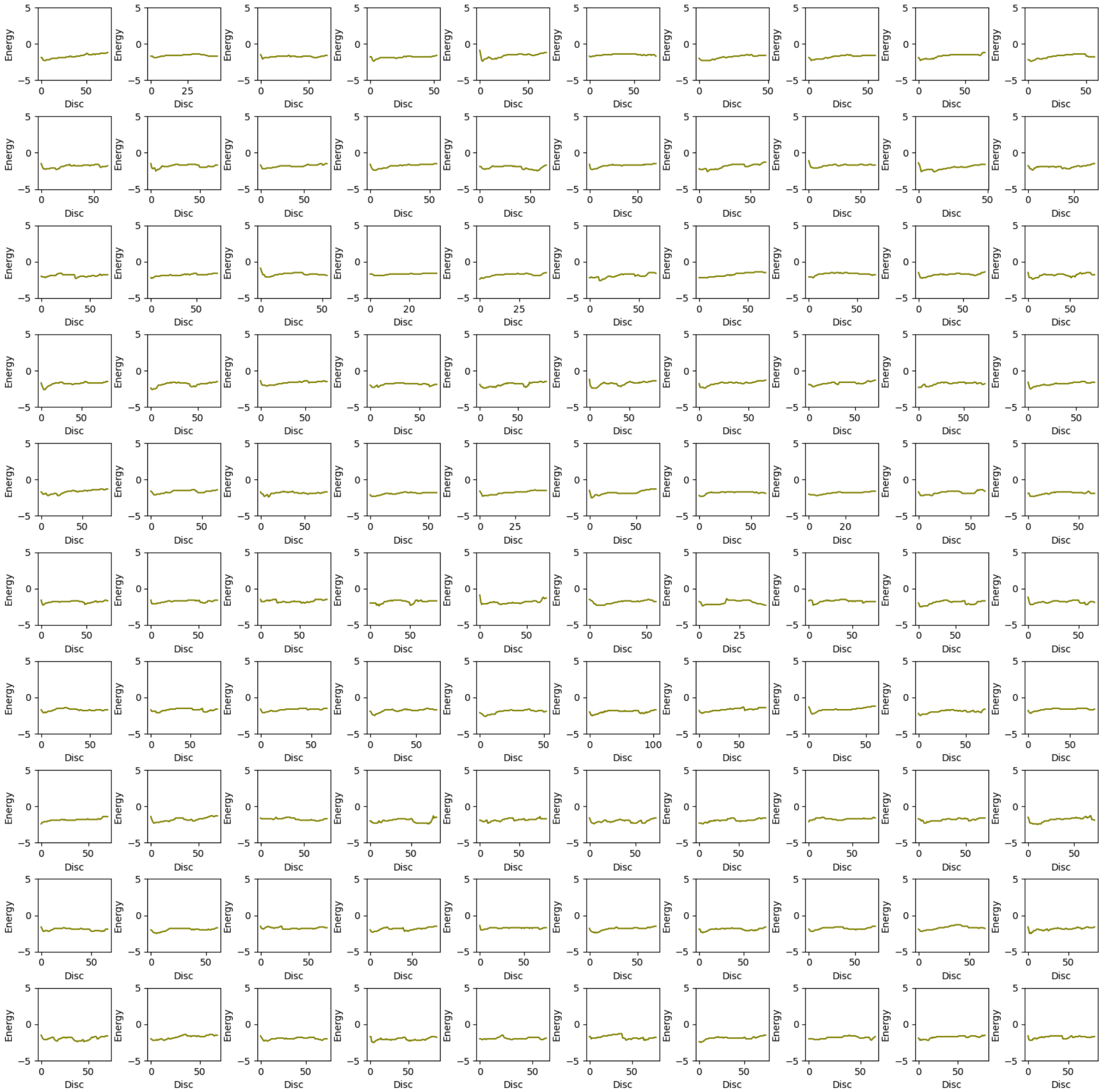

11. Upper Bound energy profile of p1b-Open with 1,2- Dibromoethane (dbe) ligand. The X-axis represents upper bound energy (kcal/mol) and Y-axis represents length of the trajectory [Å], along the disc of tunnel.

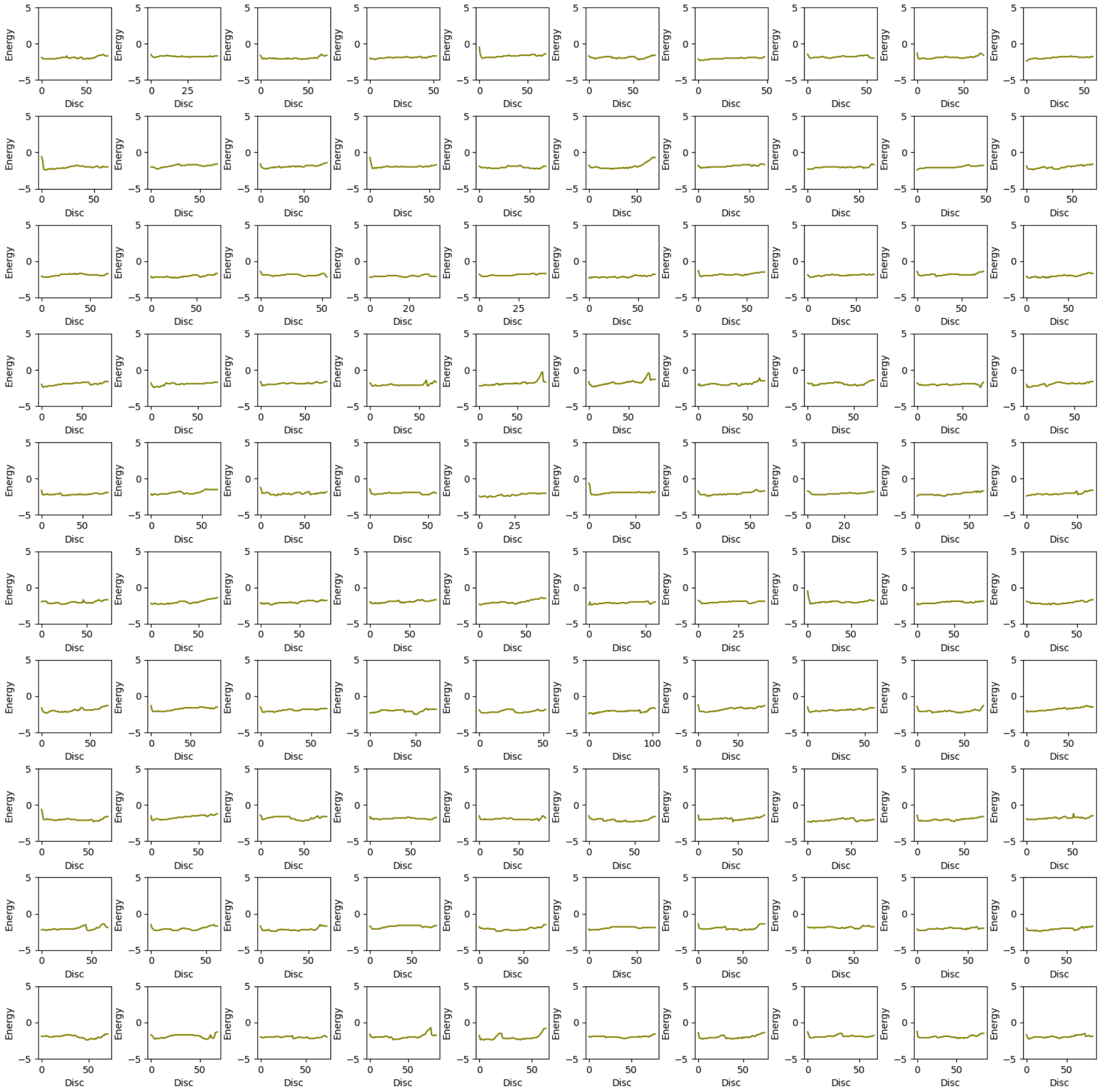

12. Upper Bound energy profile of p1b-Open with Water (H_2O) ligand. The X-axis represents upper bound energy (kcal/mol) and Y-axis represents length of the trajectory [Å], along the disc of tunnel.

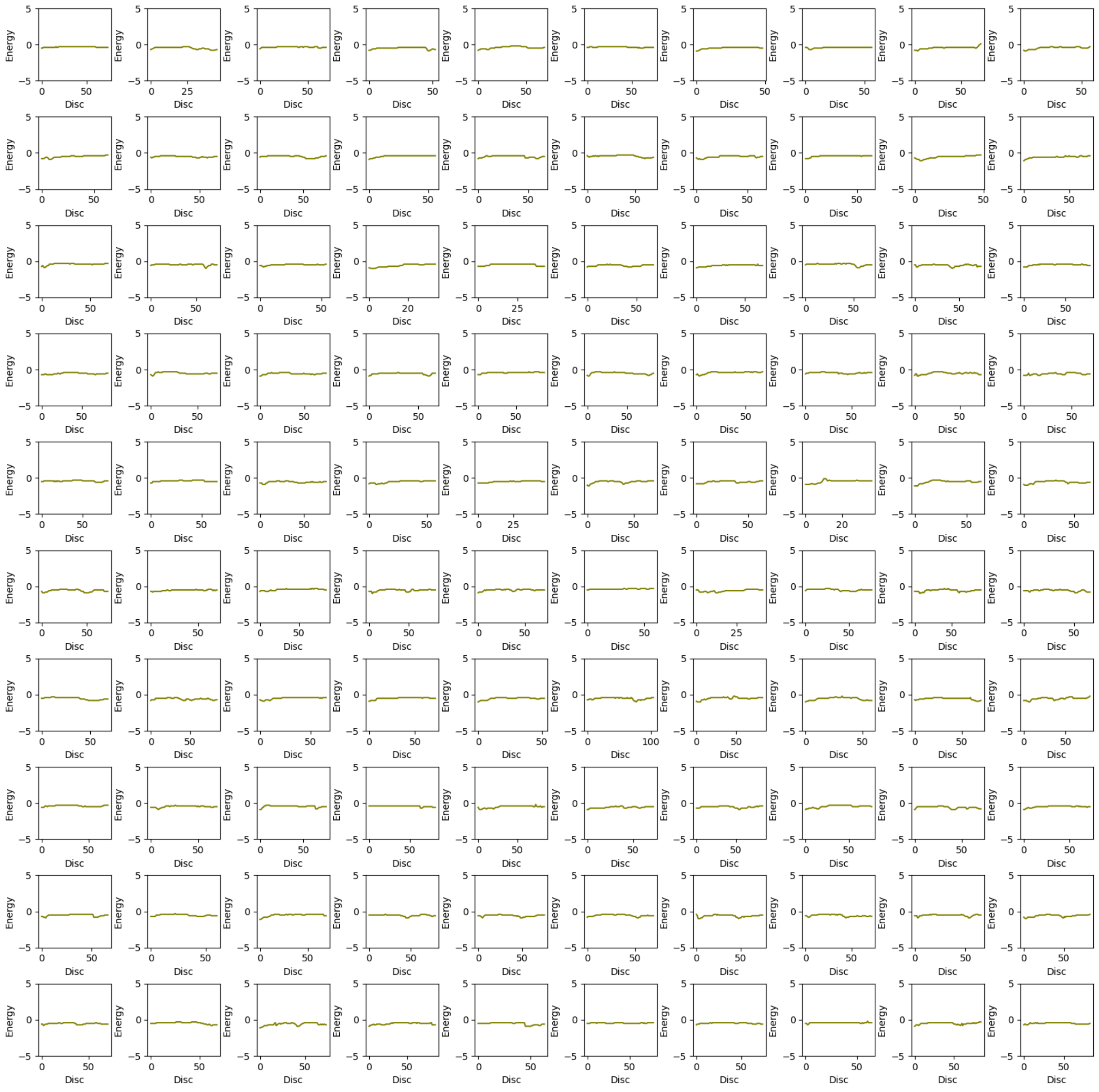

13. Upper Bound energy profile of p3-Wt with Bromide ion (Br^-) ligand. The X-axis represents upper bound energy (kcal/mol) and Y-axis represents length of the trajectory [Å], along the disc of tunnel.

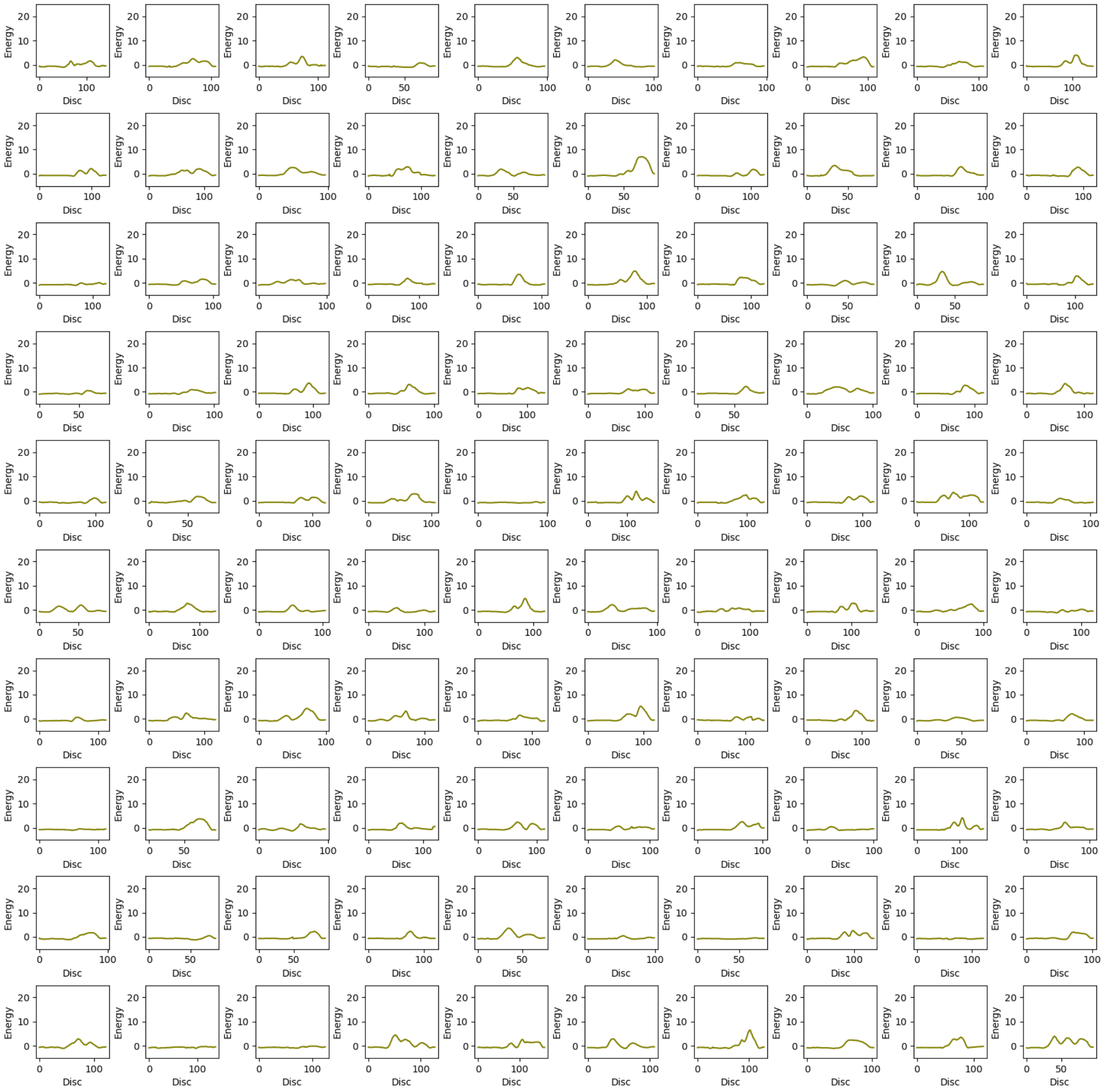

14. Upper Bound energy profile of p3-Wt with 2-Bromoethanol (be) ligand. The X-axis represents upper bound energy (kcal/mol) and Y-axis represents length of the trajectory [Å], along the disc of tunnel.

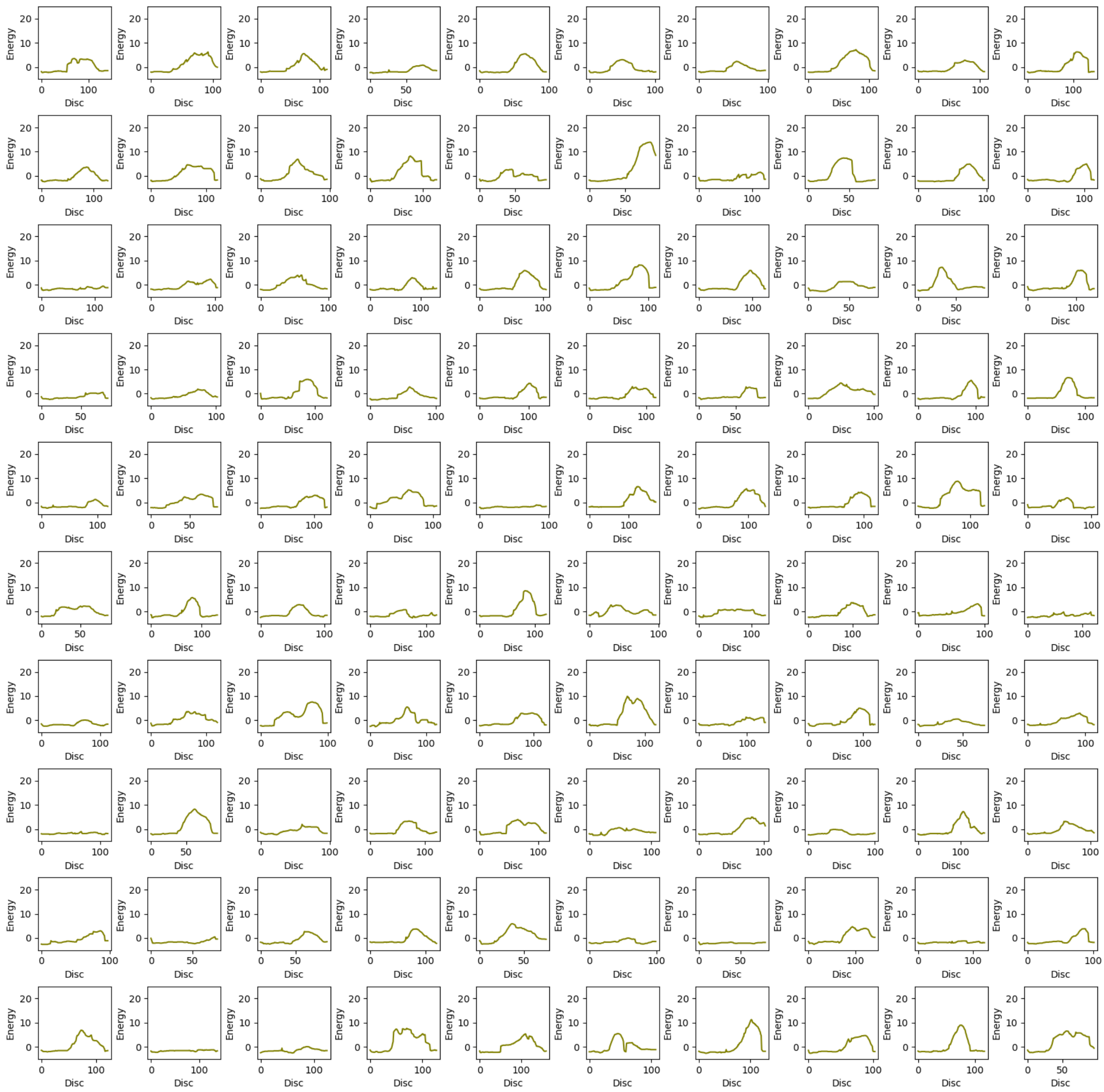

15. Upper Bound energy profile of p3-Wt with 1,2- Dibromoethane (dbe) ligand. The X-axis represents upper bound energy (kcal/mol) and Y-axis represents length of the trajectory [Å], along the disc of tunnel.

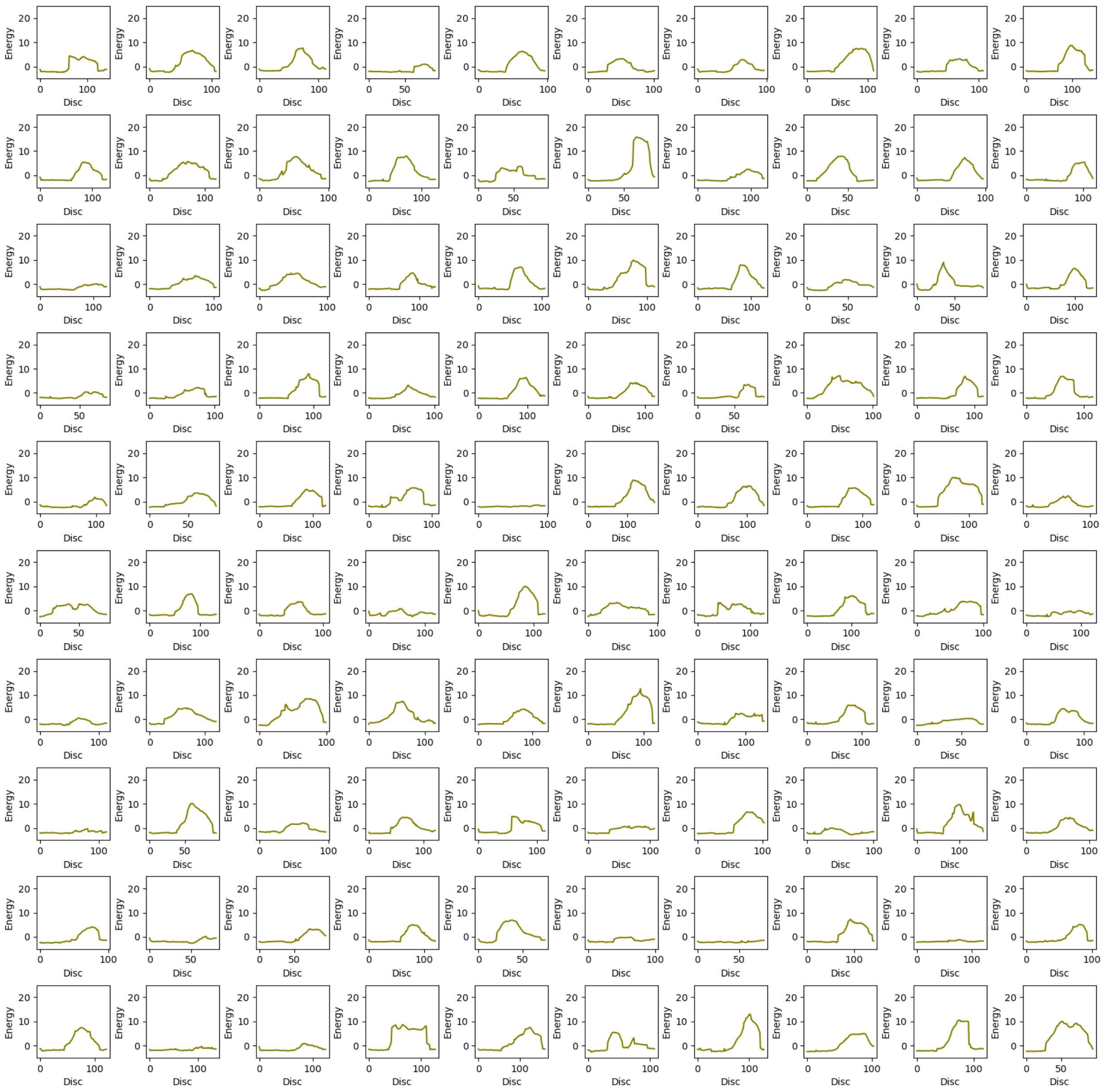

16. Upper Bound energy profile of p3-Wt with Water (H_2O) ligand. The X-axis represents upper bound energy (kcal/mol) and Y-axis represents length of the trajectory [Å], along the disc of tunnel.

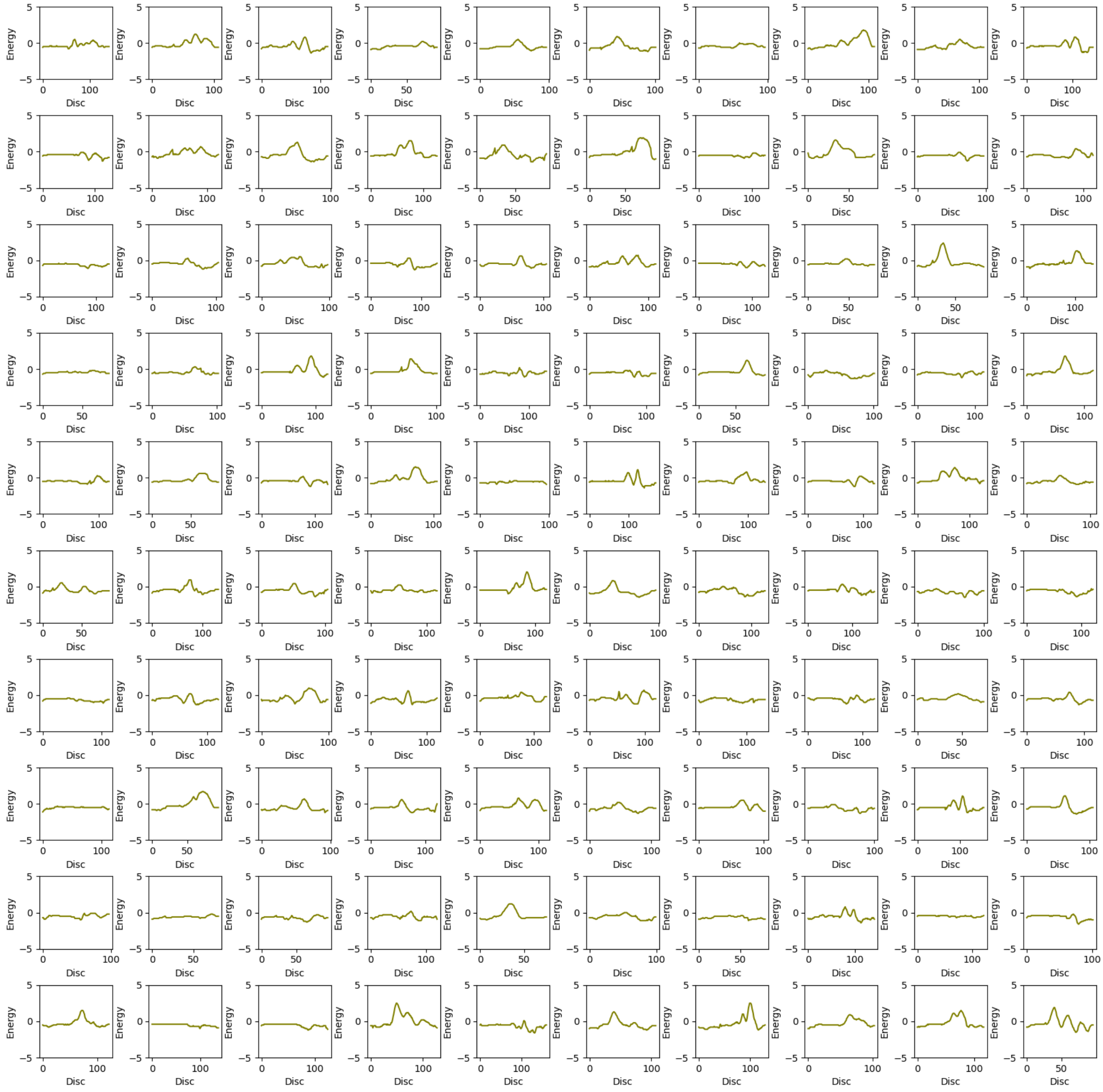

17. Upper Bound energy profile of p3-Closed with Bromide ion (Br^-) ligand. The X-axis represents upper bound energy (kcal/mol) and Y-axis represents length of the trajectory [Å], along the disc of tunnel.

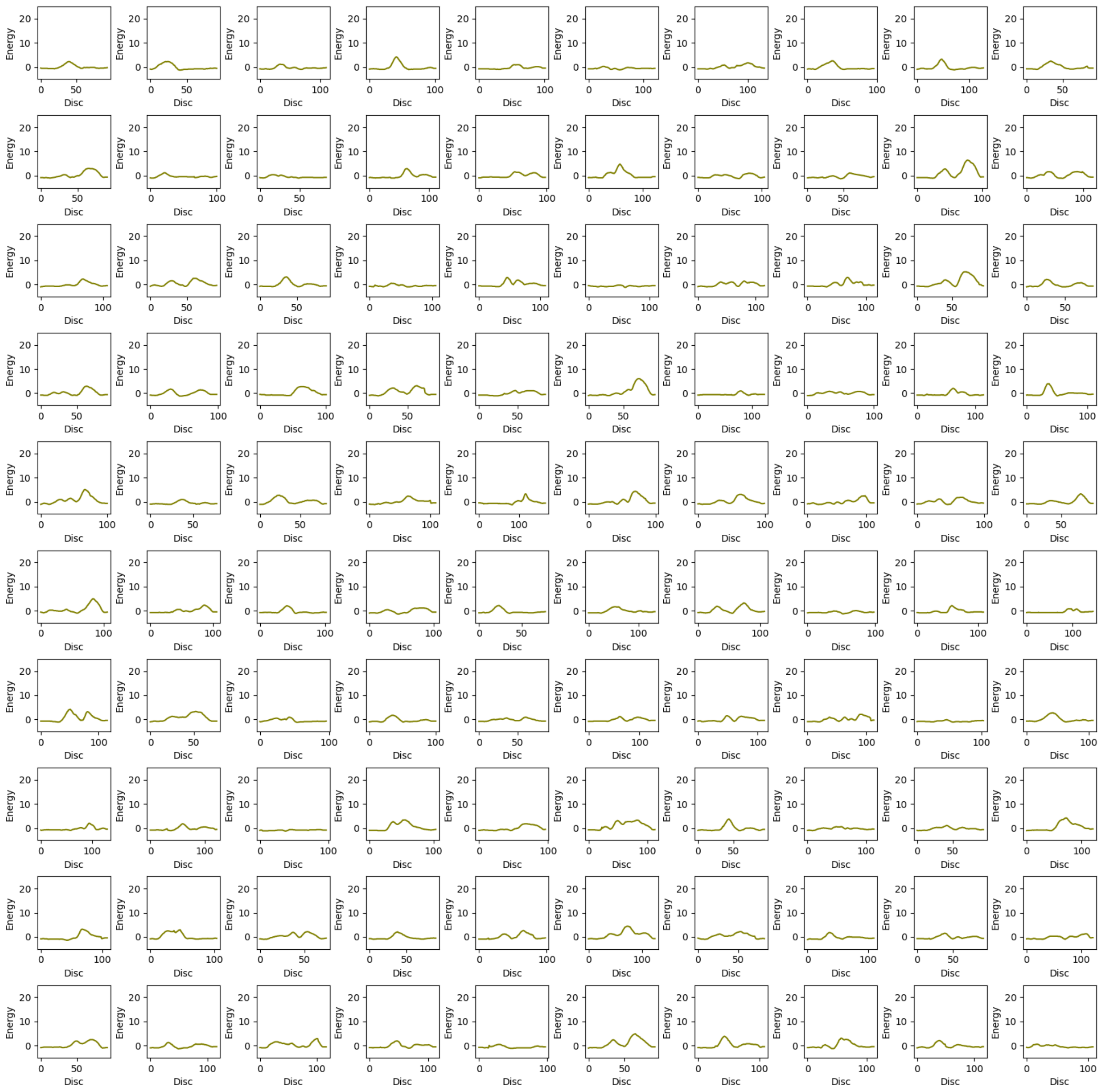

18. Upper Bound energy profile of p3-Closed with 2-Bromoethanol (be) ligand. The X-axis represents upper bound energy (kcal/mol) and Y-axis represents length of the trajectory [Å], along the disc of tunnel.

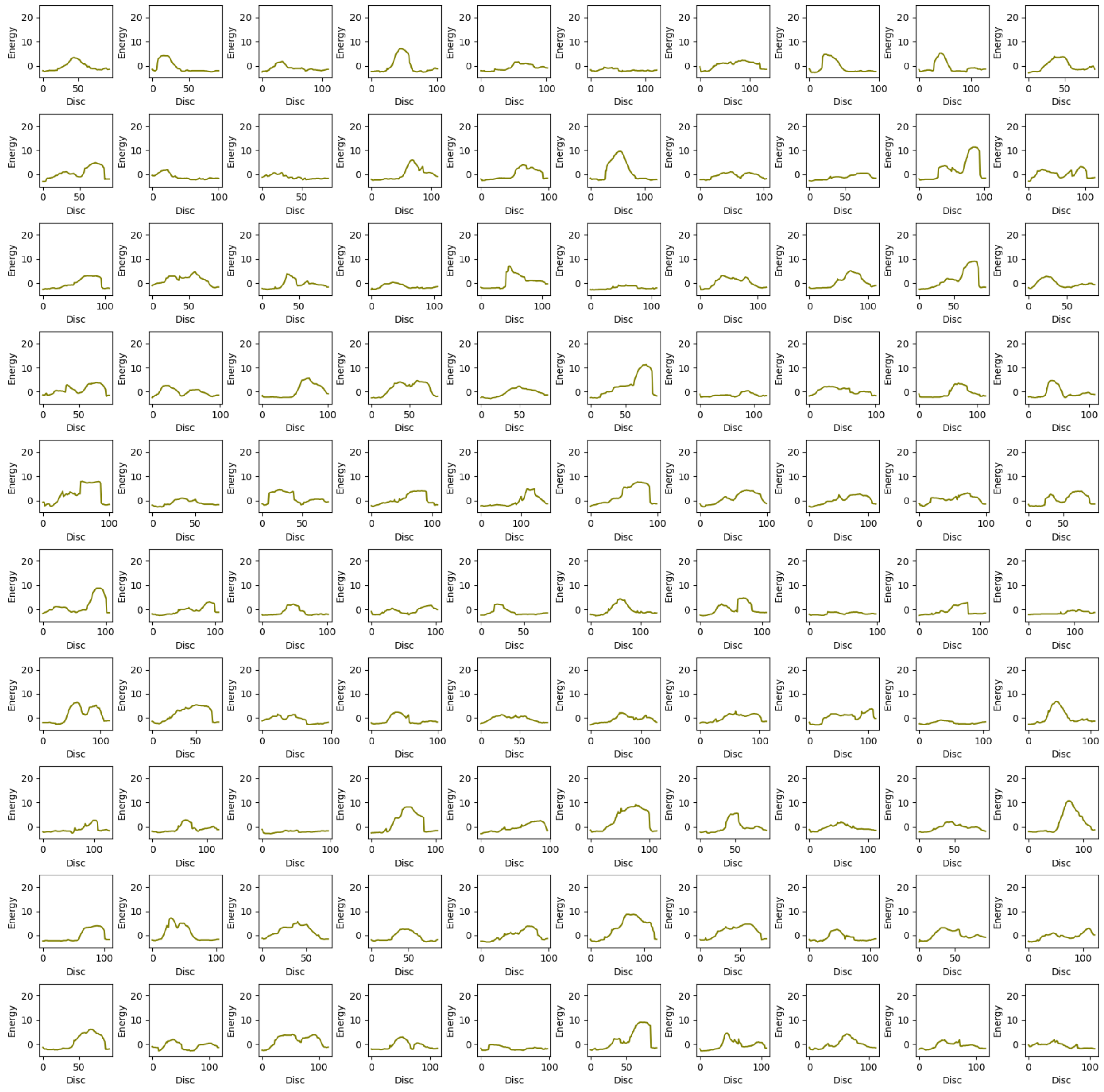

19. Upper Bound energy profile of p3-Closed with 1,2- Dibromoethane (dbe) ligand. The X-axis represents upper bound energy (kcal/mol) and Y-axis represents length of the trajectory [Å], along the disc of tunnel.

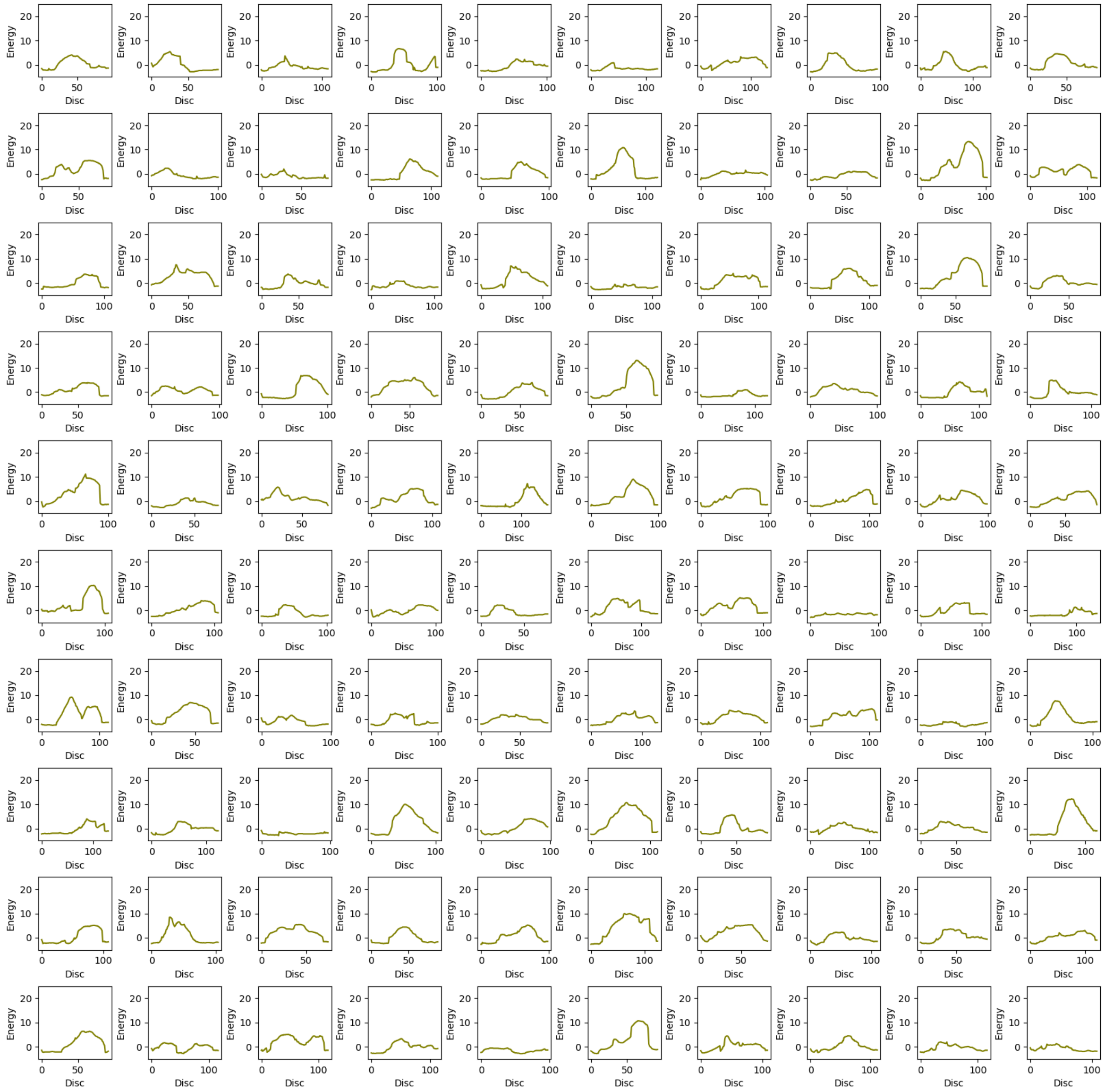

20. Upper Bound energy profile of p3-Closed with Water (H_2O) ligand. The X-axis represents upper bound energy (kcal/mol) and Y-axis represents length of the trajectory [Å], along the disc of tunnel.

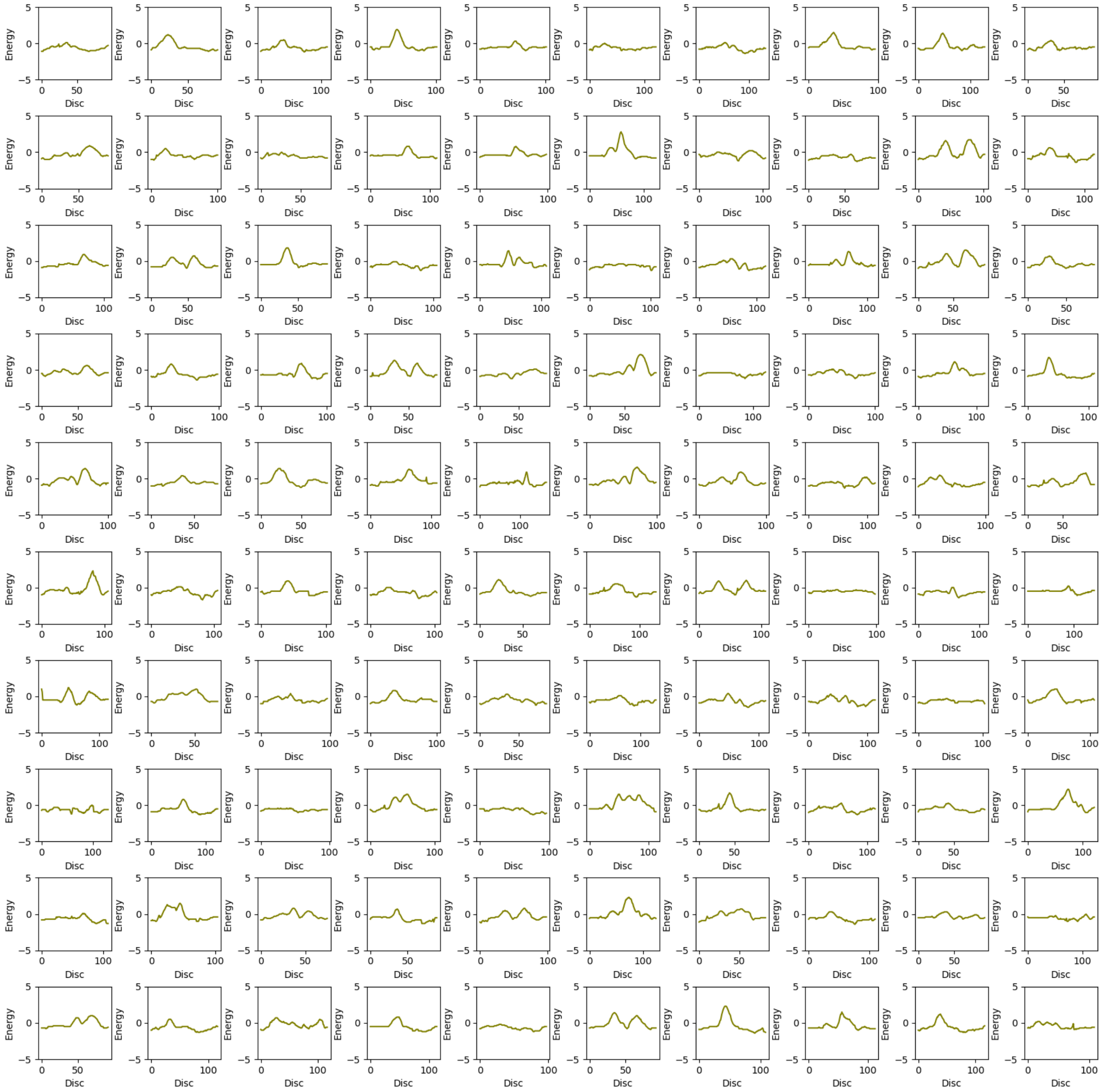

21. Upper Bound energy profile of p3-Open with Bromide ion (Br^-) ligand. The X-axis represents upper bound energy (kcal/mol) and Y-axis represents length of the trajectory [Å], along the disc of tunnel.

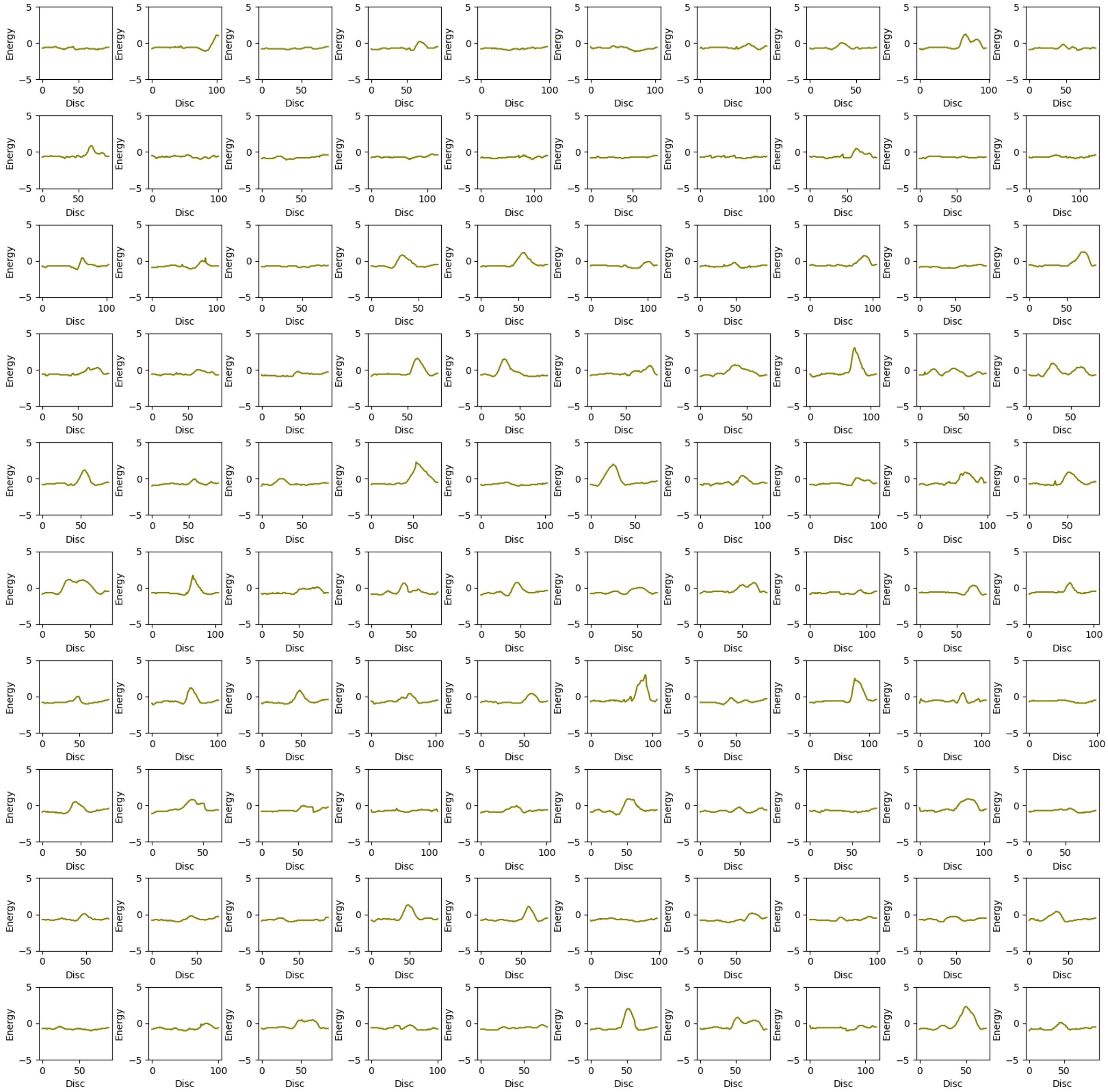

22. Upper Bound energy profile of p3-Open with 2-Bromoethanol (be) ligand. The X-axis represents upper bound energy (kcal/mol) and Y-axis represents length of the trajectory [Å], along the disc of tunnel.

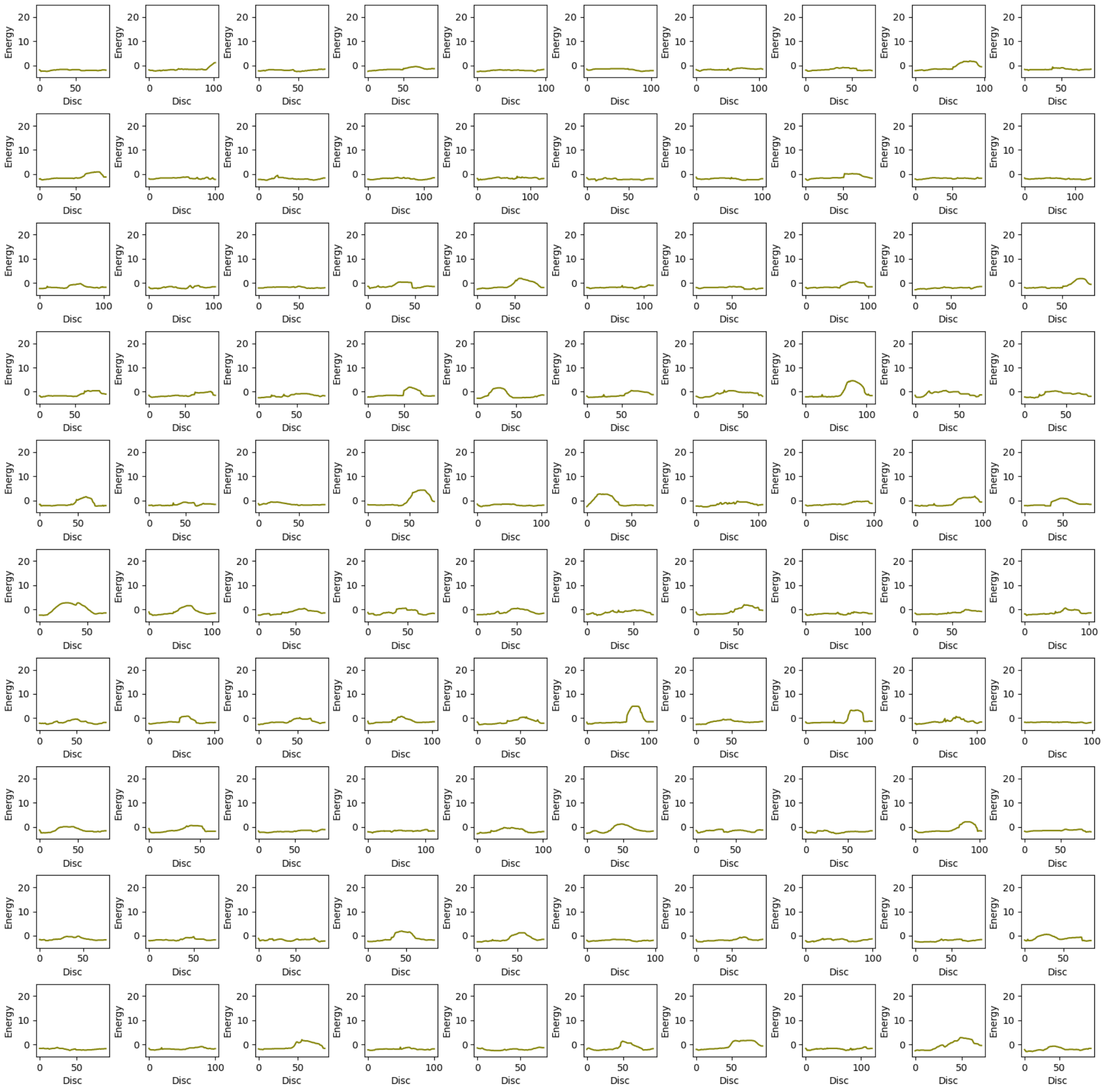

23. Upper Bound energy profile of p3-Open with 1,2- Dibromoethane (dbe) ligand. The X-axis represents upper bound energy (kcal/mol) and Y-axis represents length of the trajectory [Å], along the disc of tunnel.

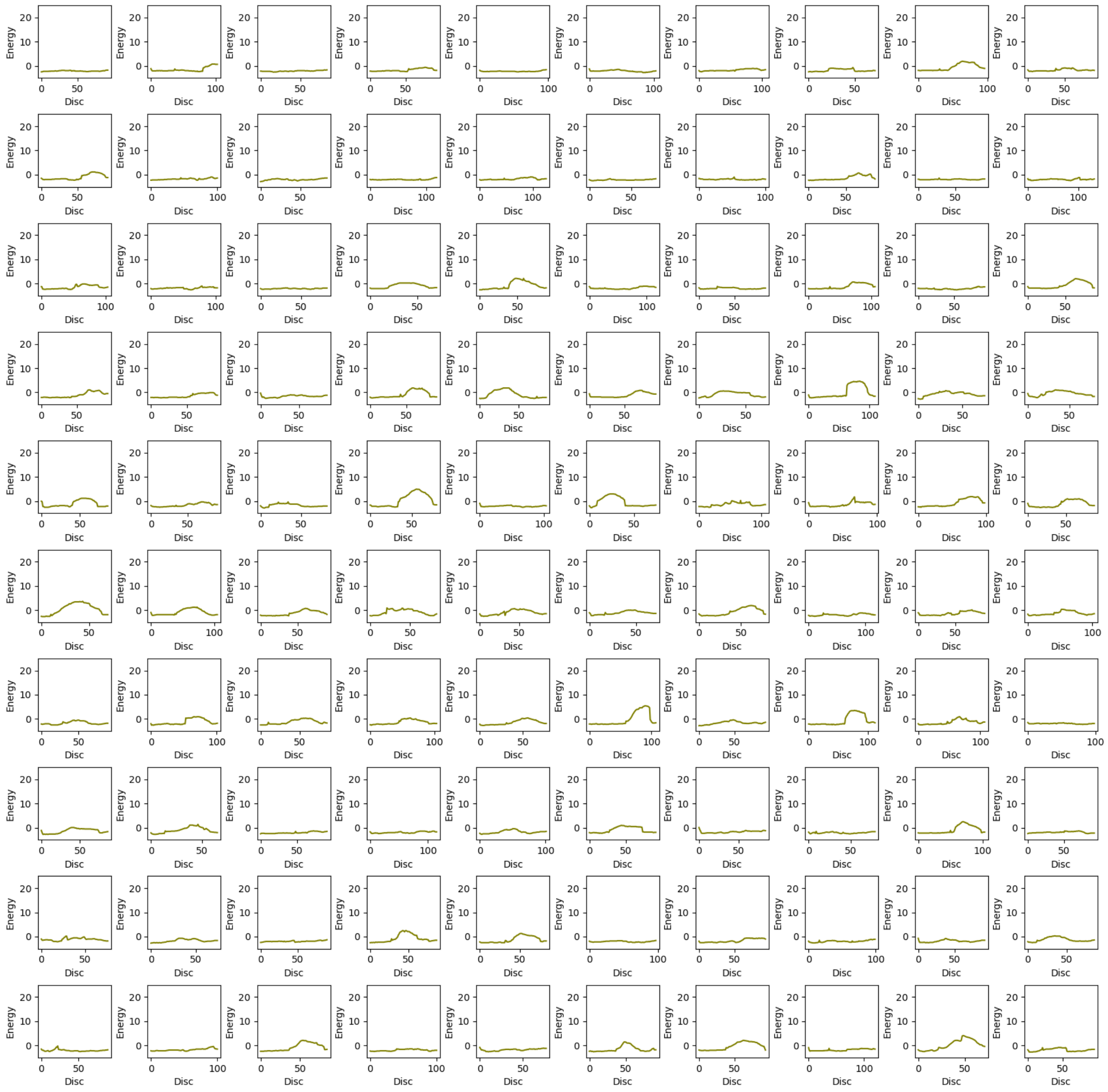

24. Upper Bound energy profile of p3-Open with Water (H_2O) ligand. The X-axis represents upper bound energy (kcal/mol) and Y-axis represents length of the trajectory [Å], along the disc of tunnel.

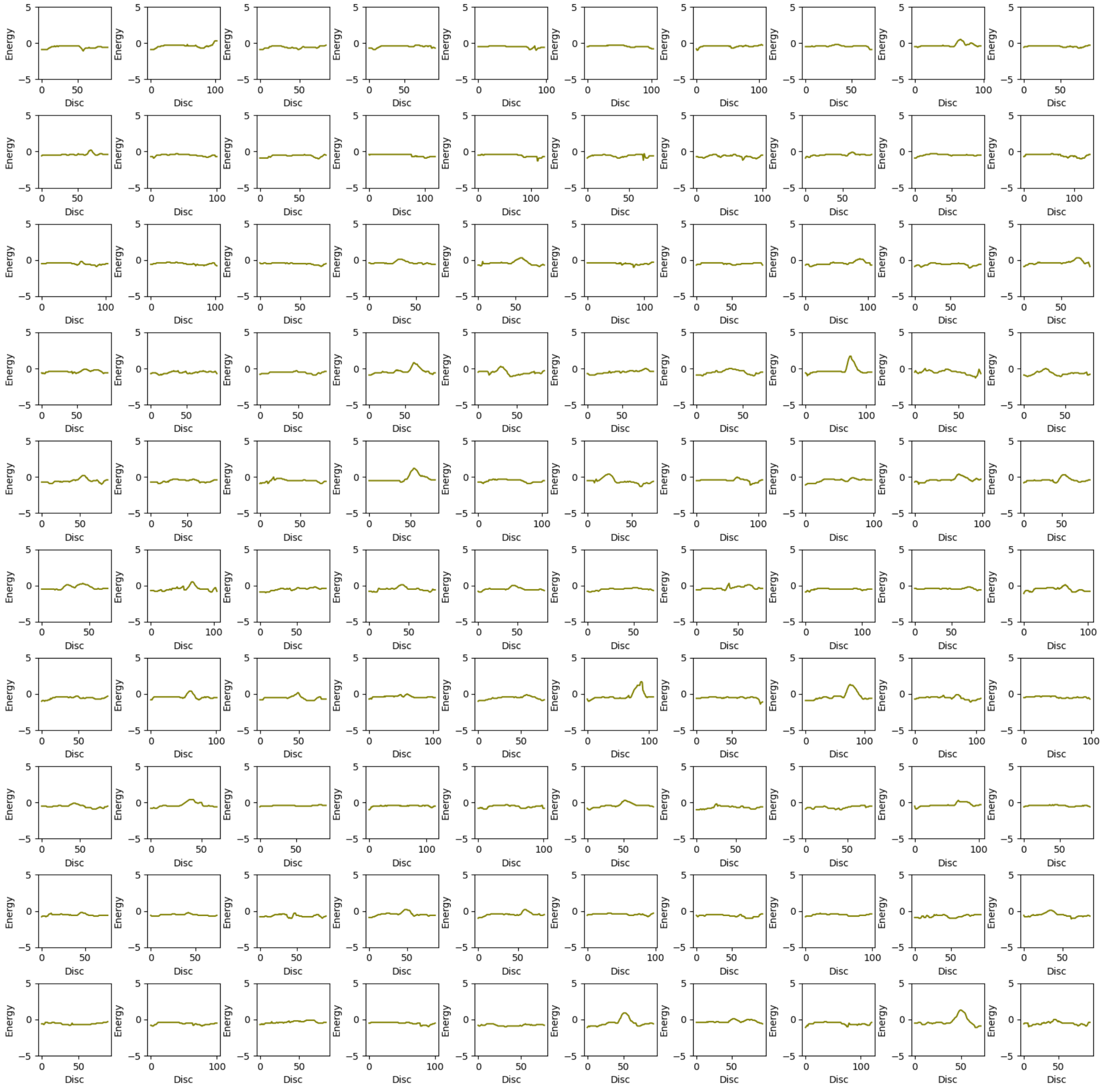

25. Upper Bound energy profile of ST-Wt with Bromide ion (Br^-) ligand. The X-axis represents upper bound energy (kcal/mol) and Y-axis represents length of the trajectory [Å], along the disc of tunnel.

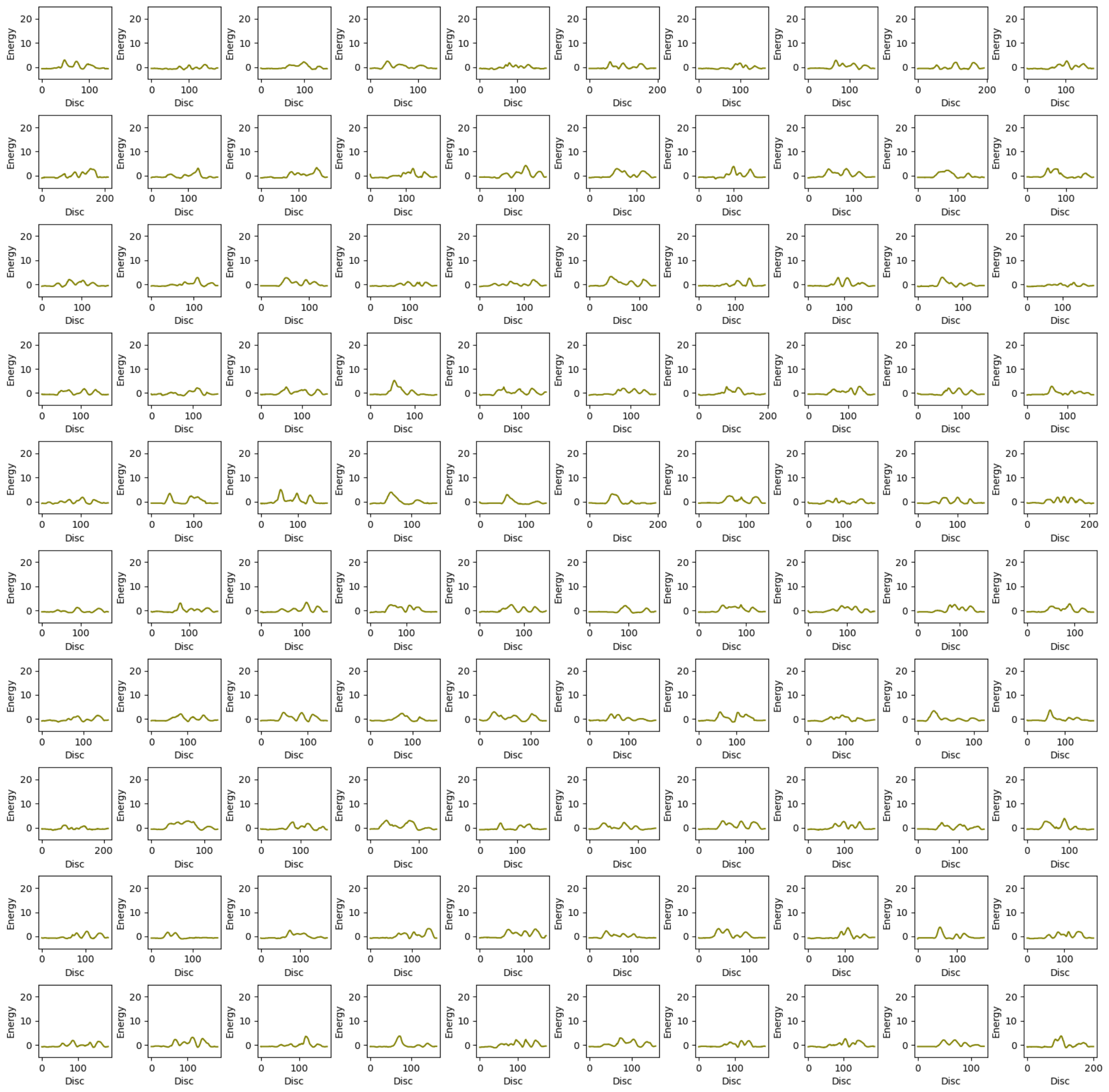

26. Upper Bound energy profile of ST-Wt with 2-Bromoethanol (be) ligand. The X-axis represents upper bound energy (kcal/mol) and Y-axis represents length of the trajectory [Å], along the disc of tunnel.

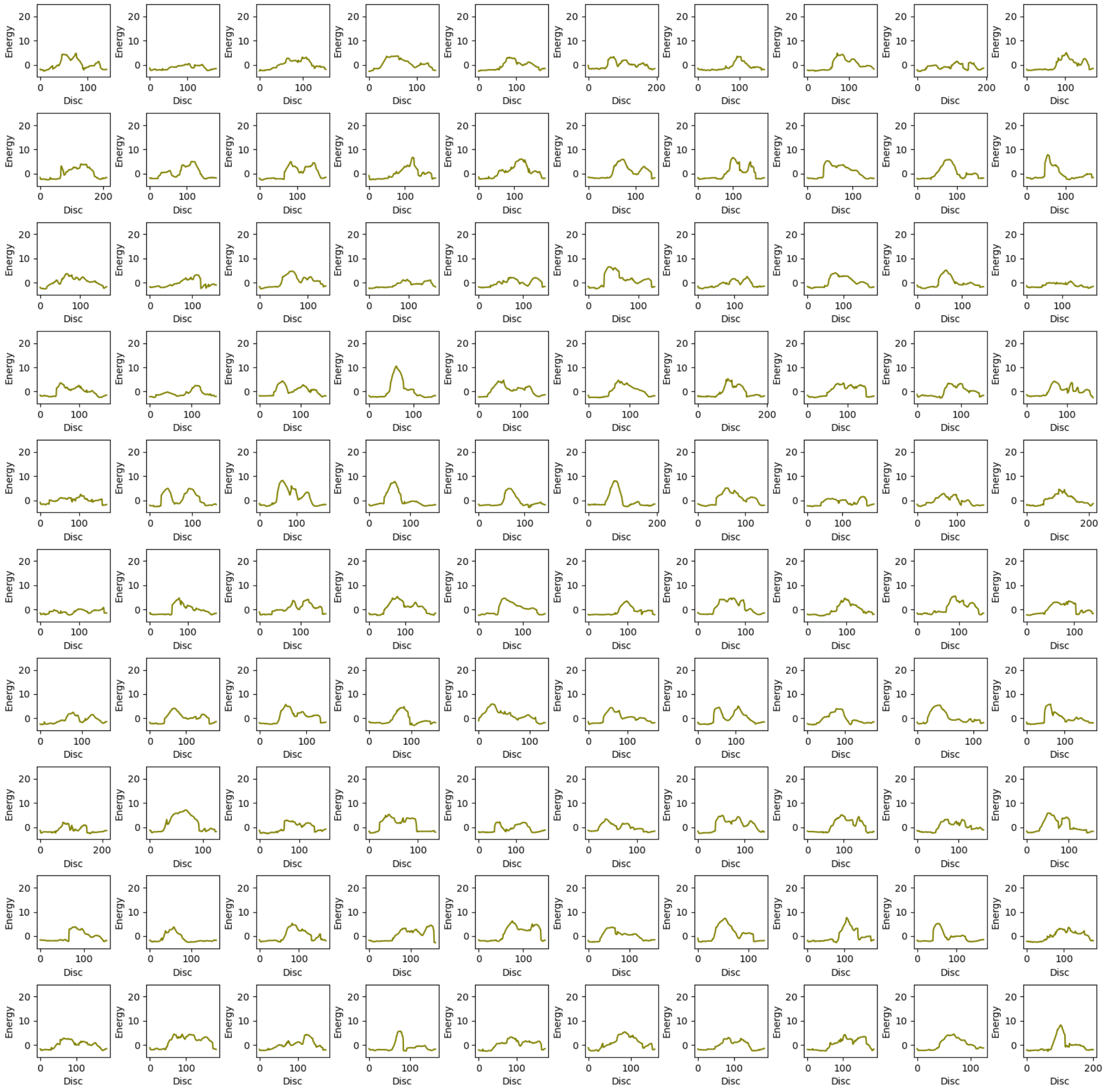

27. Upper Bound energy profile of ST-Wt with 1,2- Dibromoethane (dbe) ligand. The X-axis represents upper bound energy (kcal/mol) and Y-axis represents length of the trajectory [Å], along the disc of tunnel.

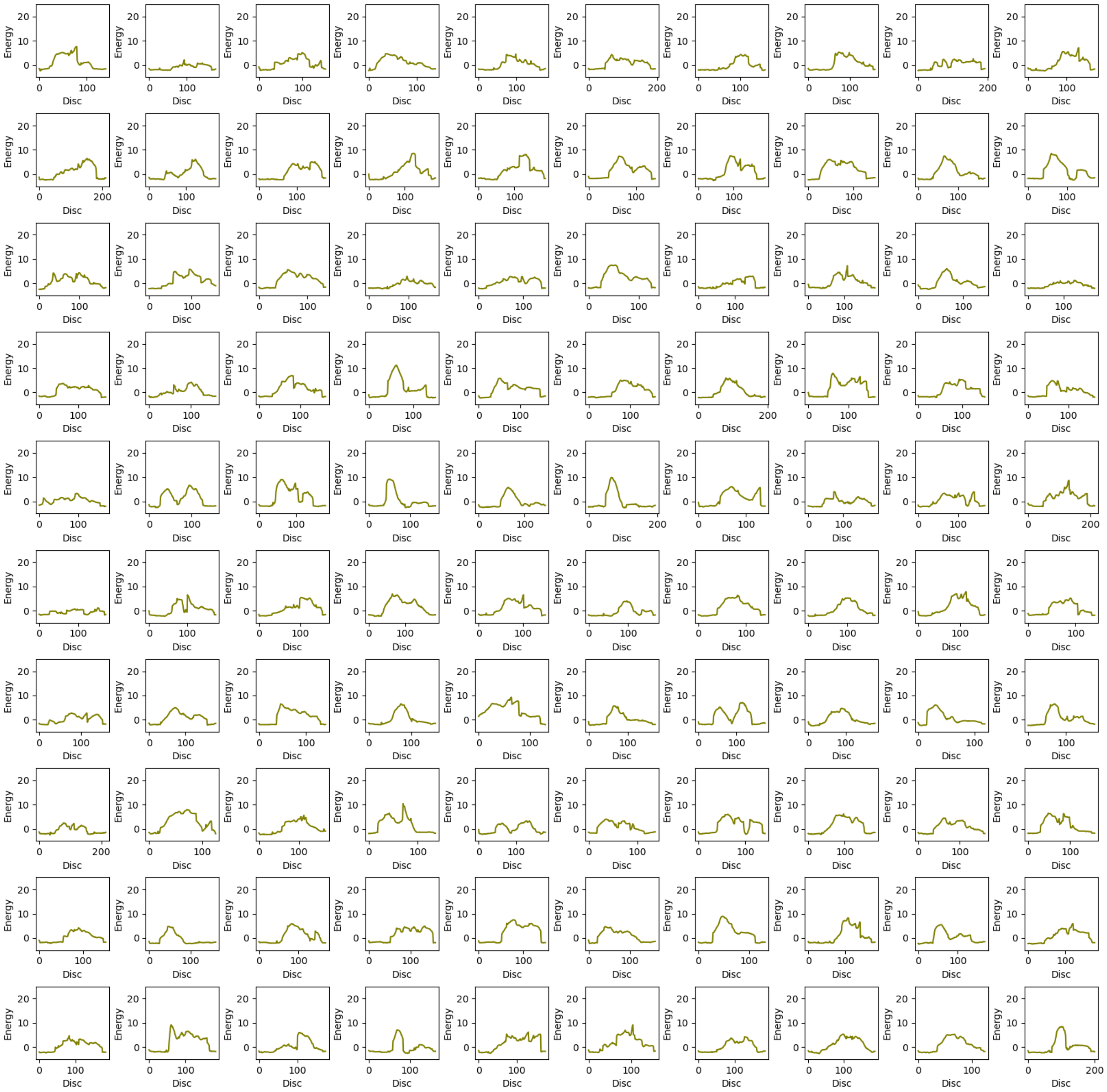

28. Upper Bound energy profile of ST-Wt with Water (H_2O) ligand. The X-axis represents upper bound energy (kcal/mol) and Y-axis represents length of the trajectory [Å], along the disc of tunnel.

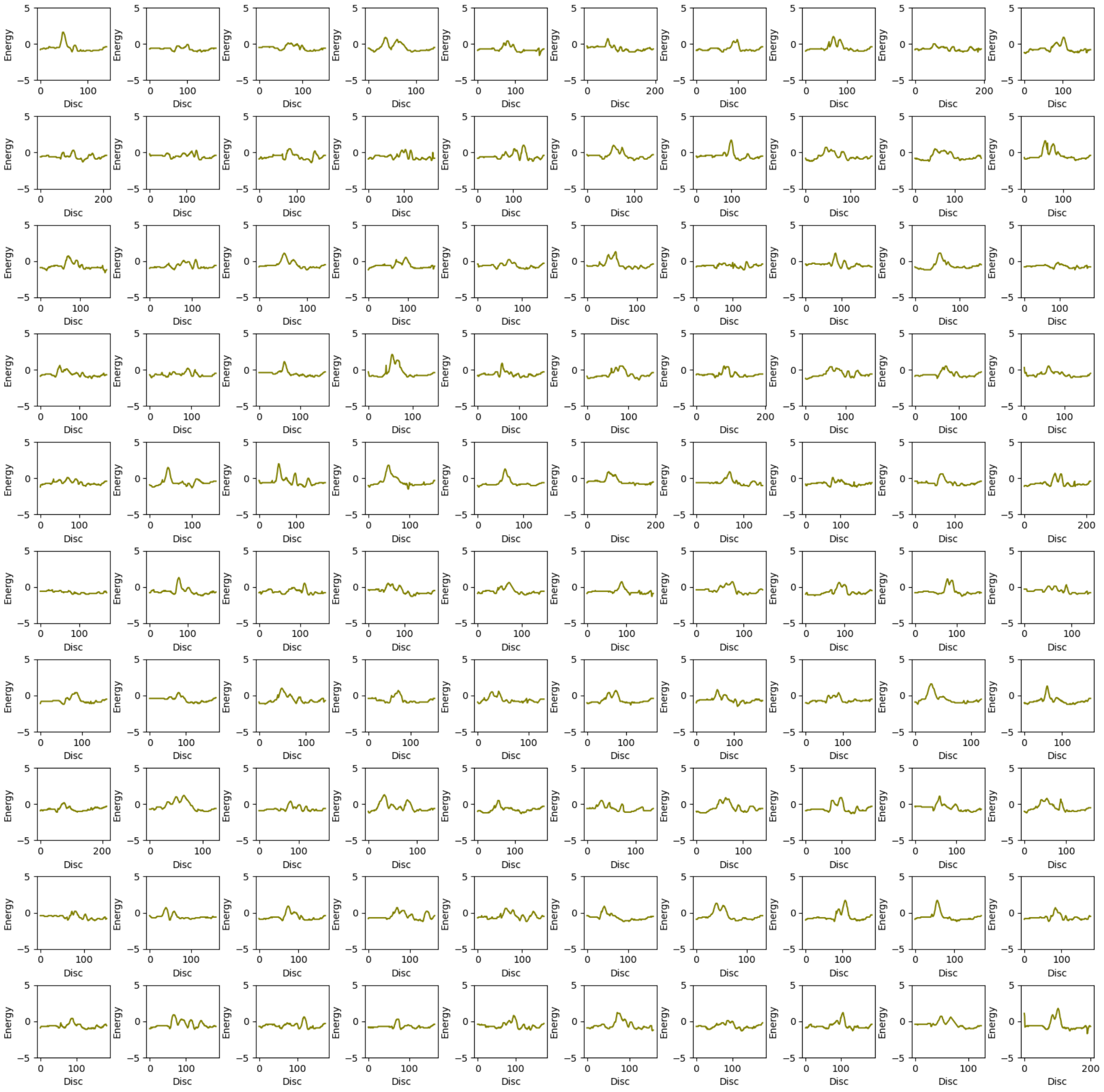

29. Upper Bound energy profile of ST-Closed with Bromide ion (Br^-) ligand. The X-axis represents upper bound energy (kcal/mol) and Y-axis represents length of the trajectory [Å], along the disc of tunnel.

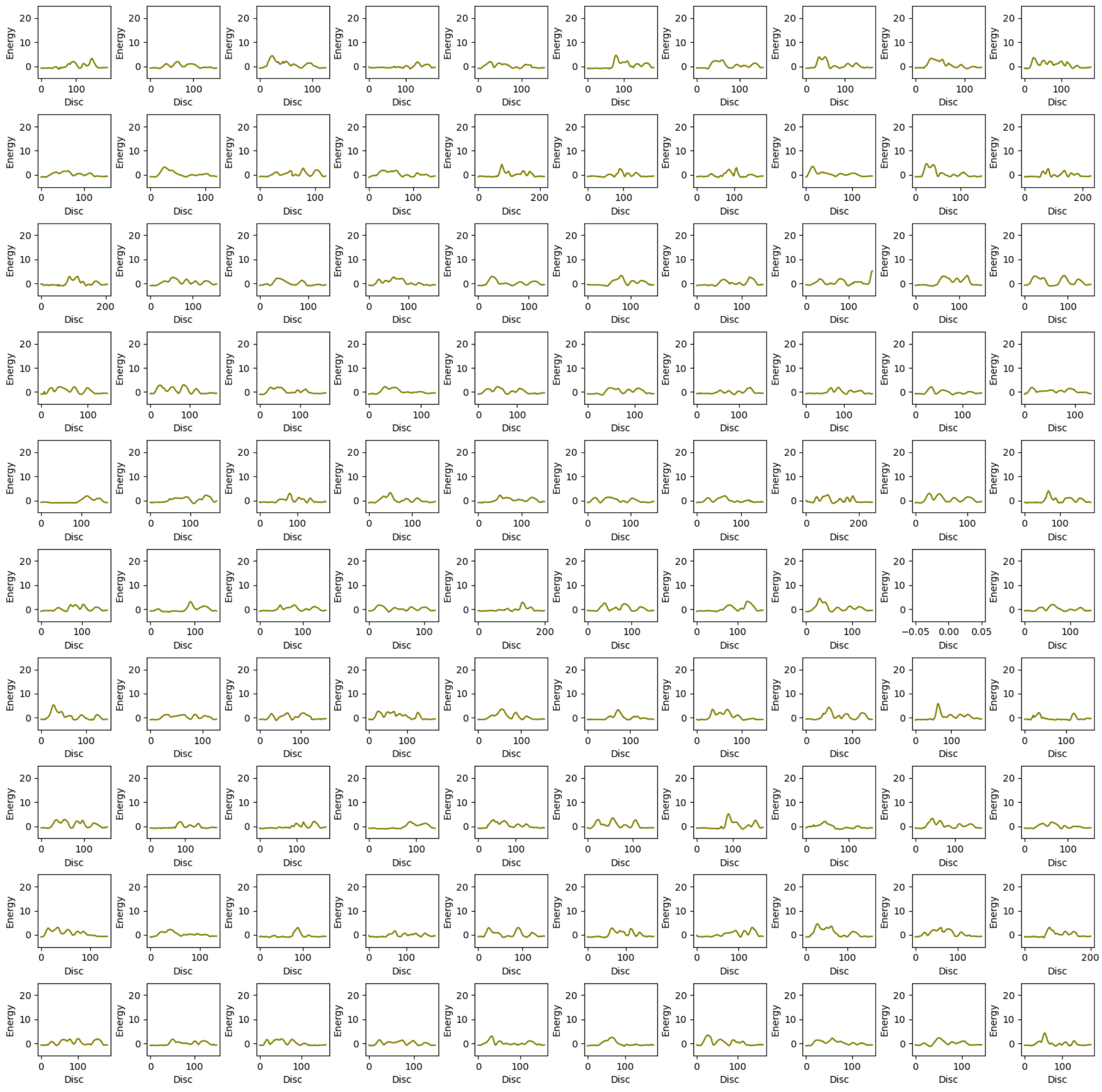

30. Upper Bound energy profile of ST-Closed with 2-Bromoethanol (be) ligand. The X-axis represents upper bound energy (kcal/mol) and Y-axis represents length of the trajectory [Å], along the disc of tunnel.

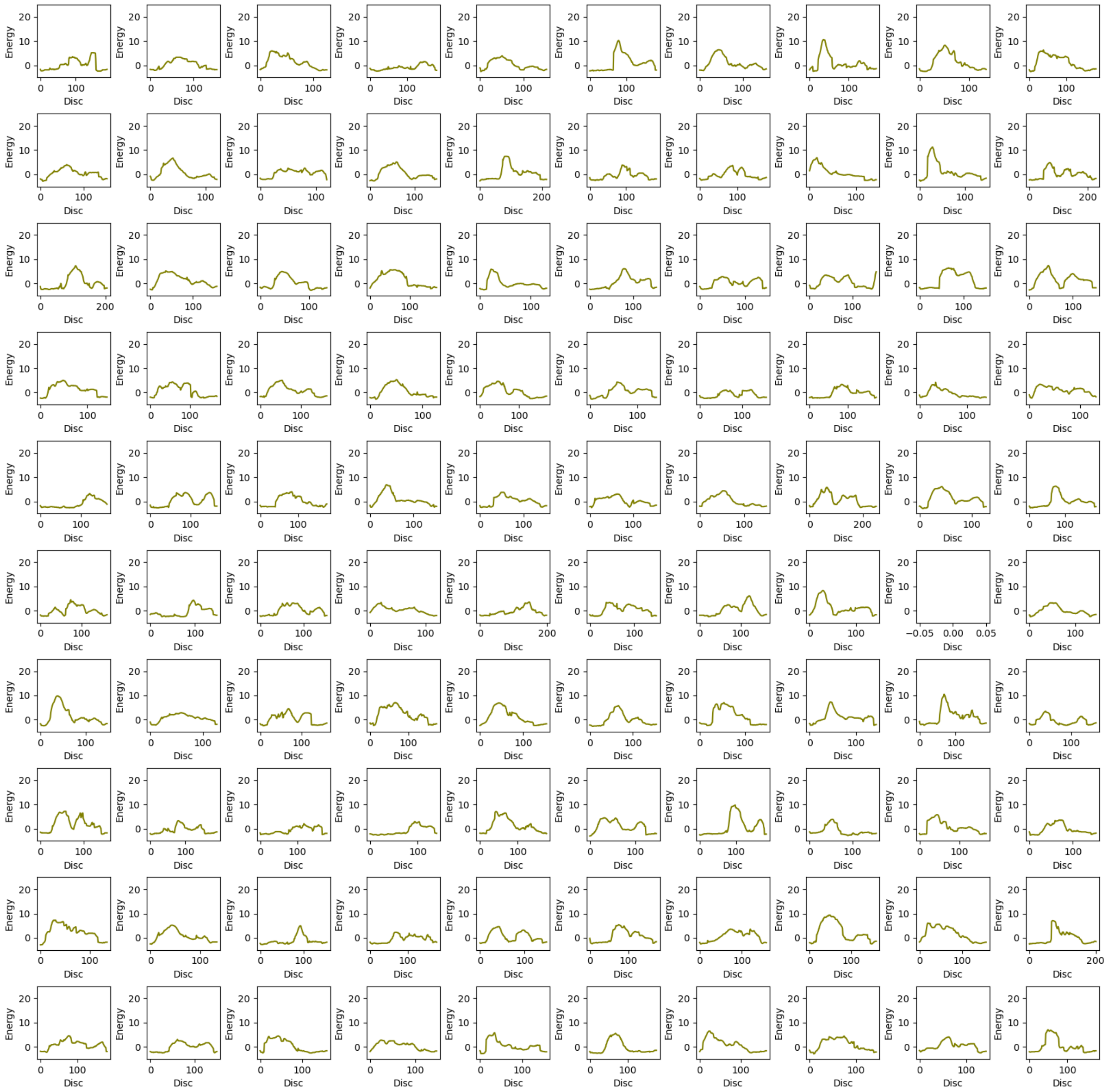

31. Upper Bound energy profile of ST-Closed with 1,2- Dibromoethane (dbe) ligand. The X-axis represents upper bound energy (kcal/mol) and Y-axis represents length of the trajectory [Å], along the disc of tunnel.

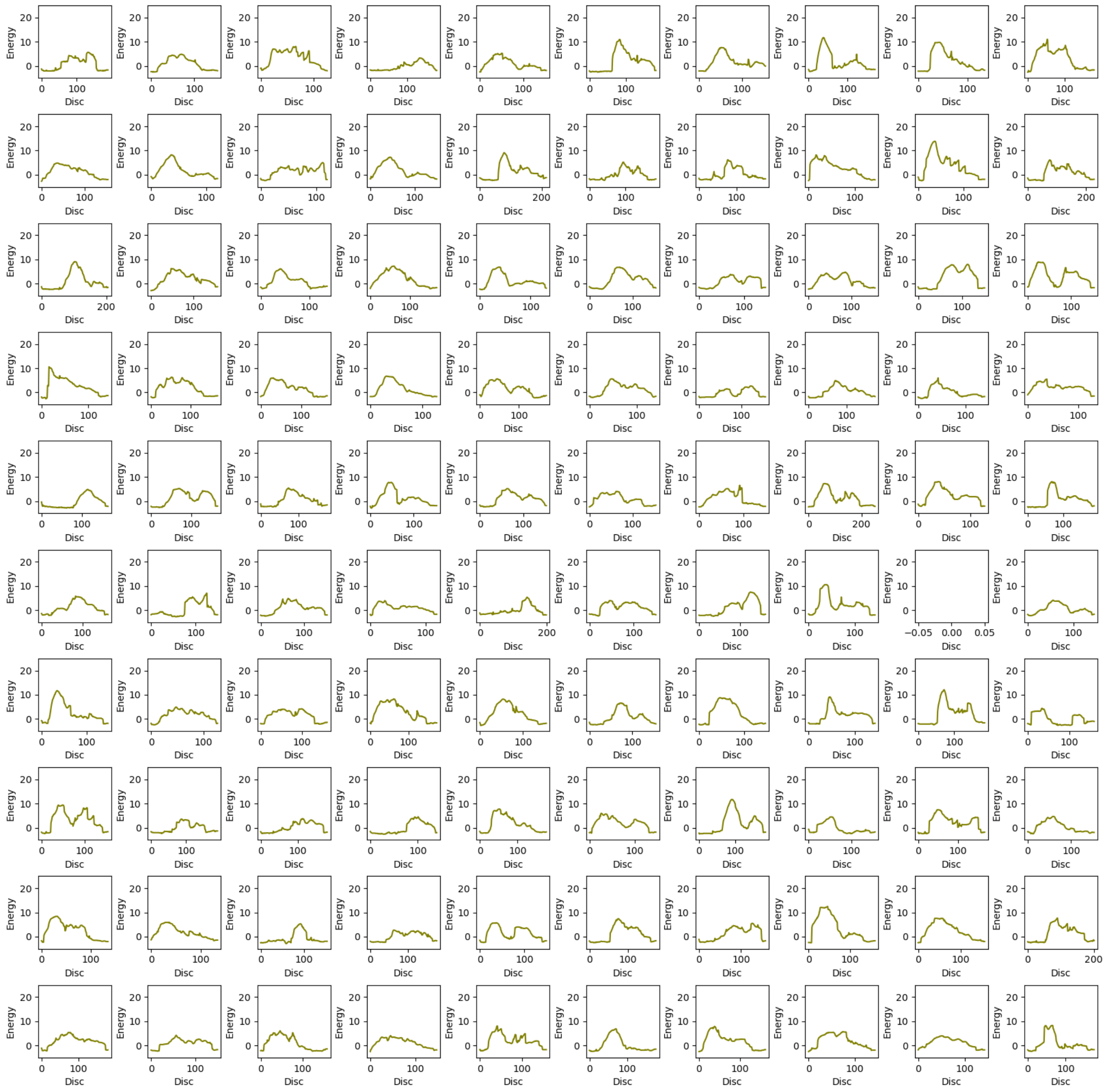

32. Upper Bound energy profile of ST-Closed with Water (H_2O) ligand. The X-axis represents upper bound energy (kcal/mol) and Y-axis represents length of the trajectory [Å], along the disc of tunnel.

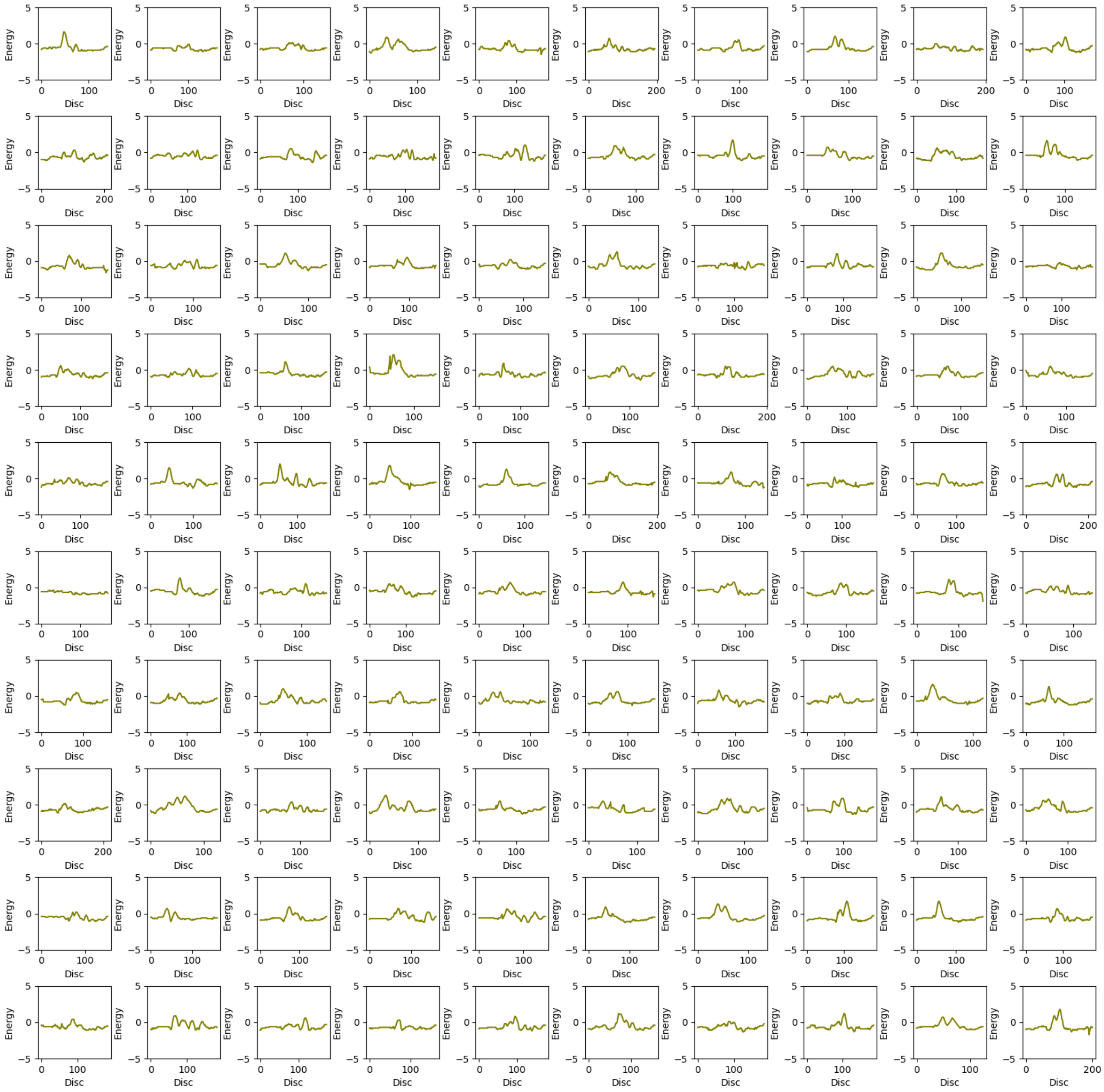

33. Upper Bound energy profile of ST-Open with Bromide ion (Br^-) ligand. The X-axis represents upper bound energy (kcal/mol) and Y-axis represents length of the trajectory [Å], along the disc of tunnel.

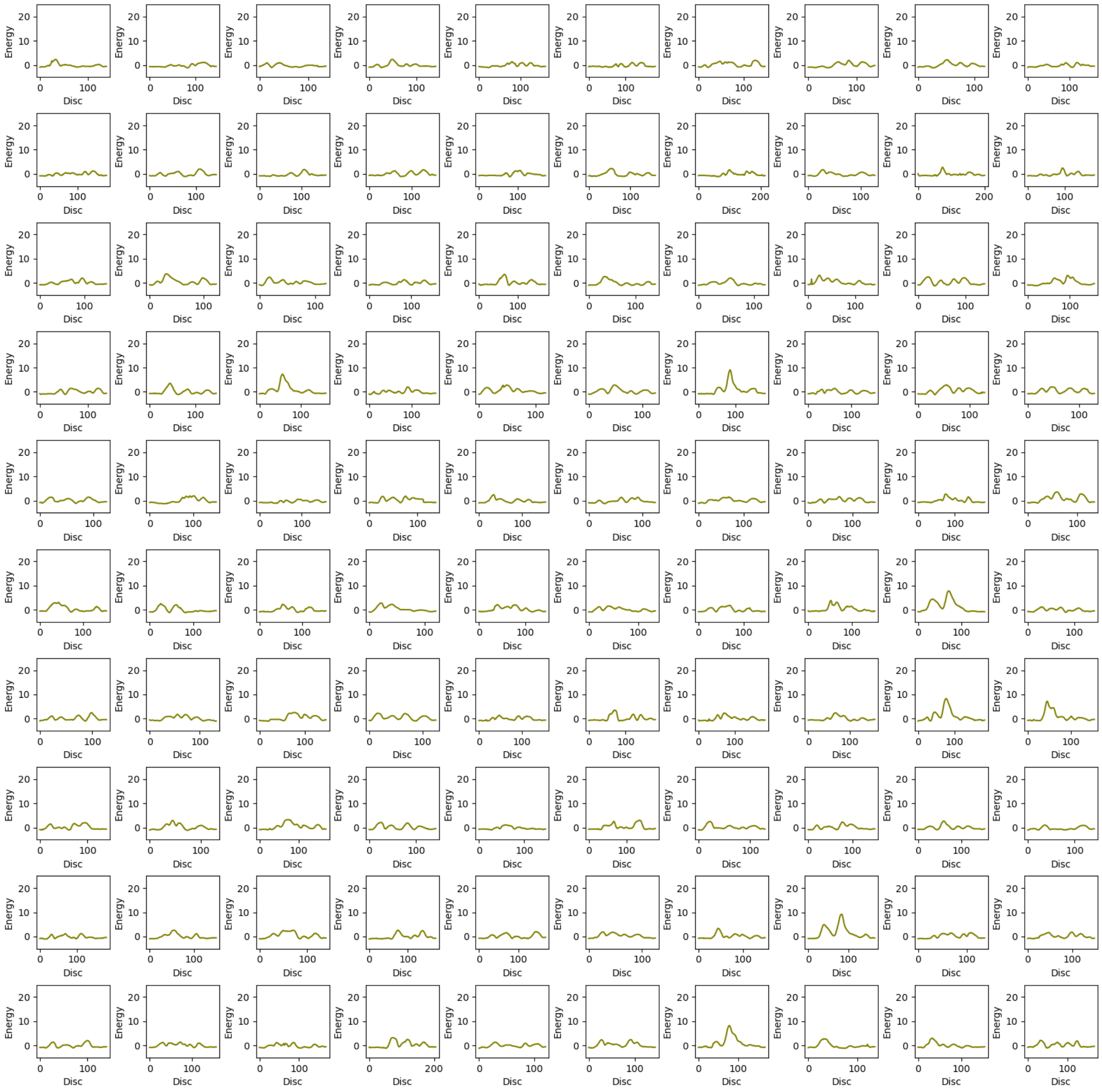

34. Upper Bound energy profile of ST-Open with 2-Bromoethanol (be) ligand. The X-axis represents upper bound energy (kcal/mol) and Y-axis represents length of the trajectory [Å], along the disc of tunnel.

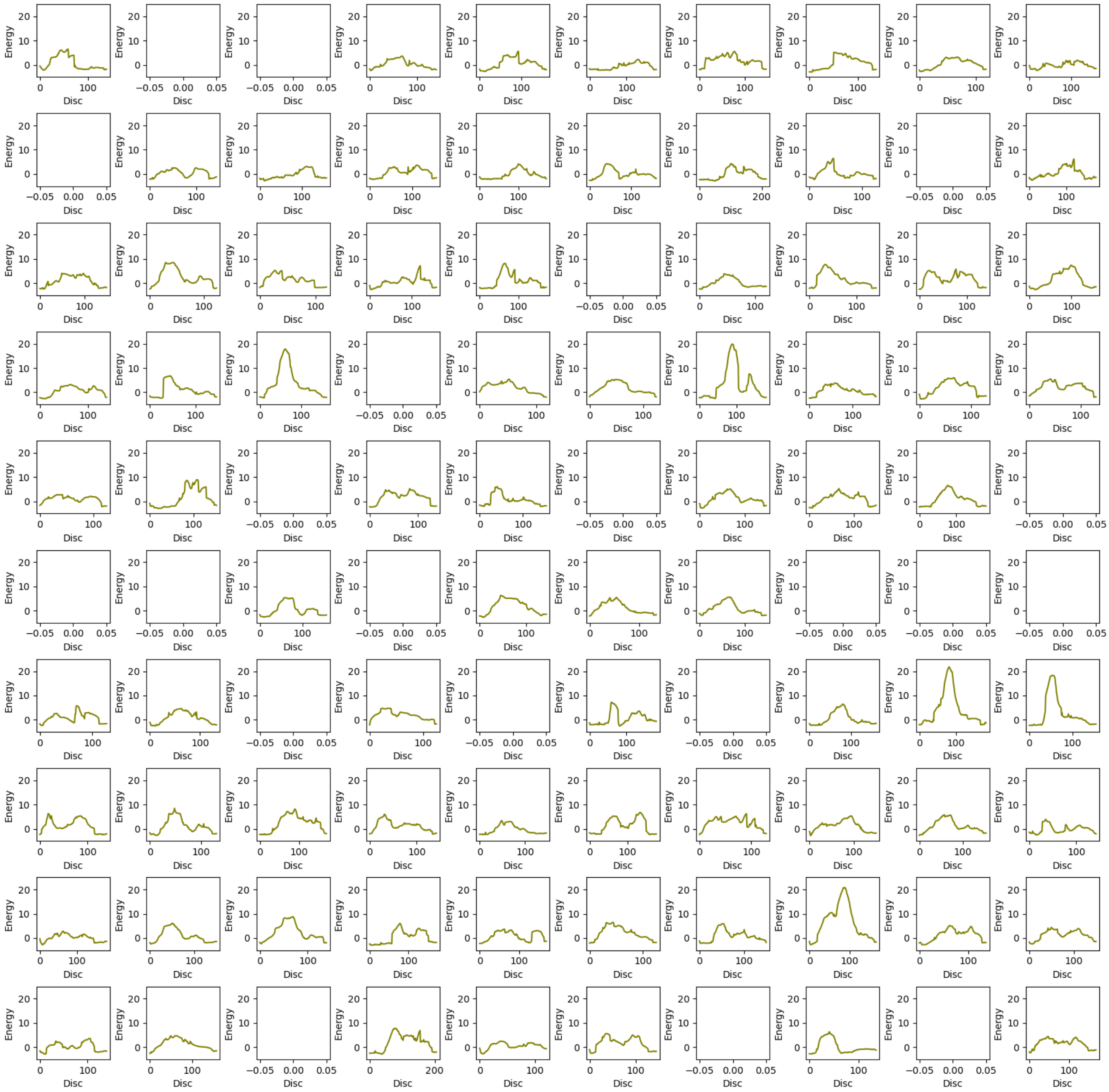

35. Upper Bound energy profile of ST-Open with 1,2- Dibromoethane (dbe) ligand. The X-axis represents upper bound energy (kcal/mol) and Y-axis represents length of the trajectory [Å], along the disc of tunnel.

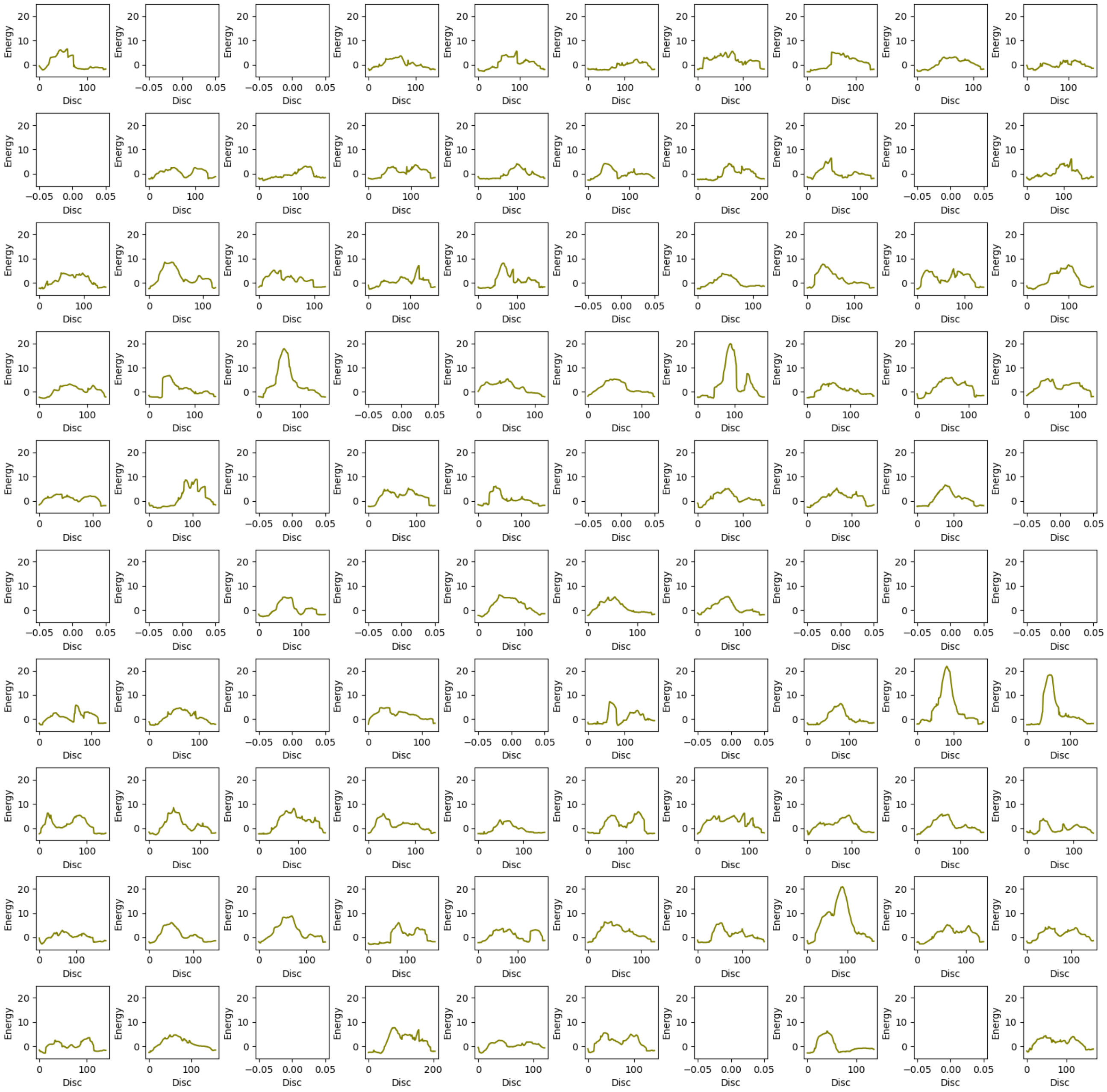

36. Upper Bound energy profile of ST-Open with Water (H_2O) ligand. The X-axis represents upper bound energy (kcal/mol) and Y-axis represents length of the trajectory [Å], along the disc of tunnel.

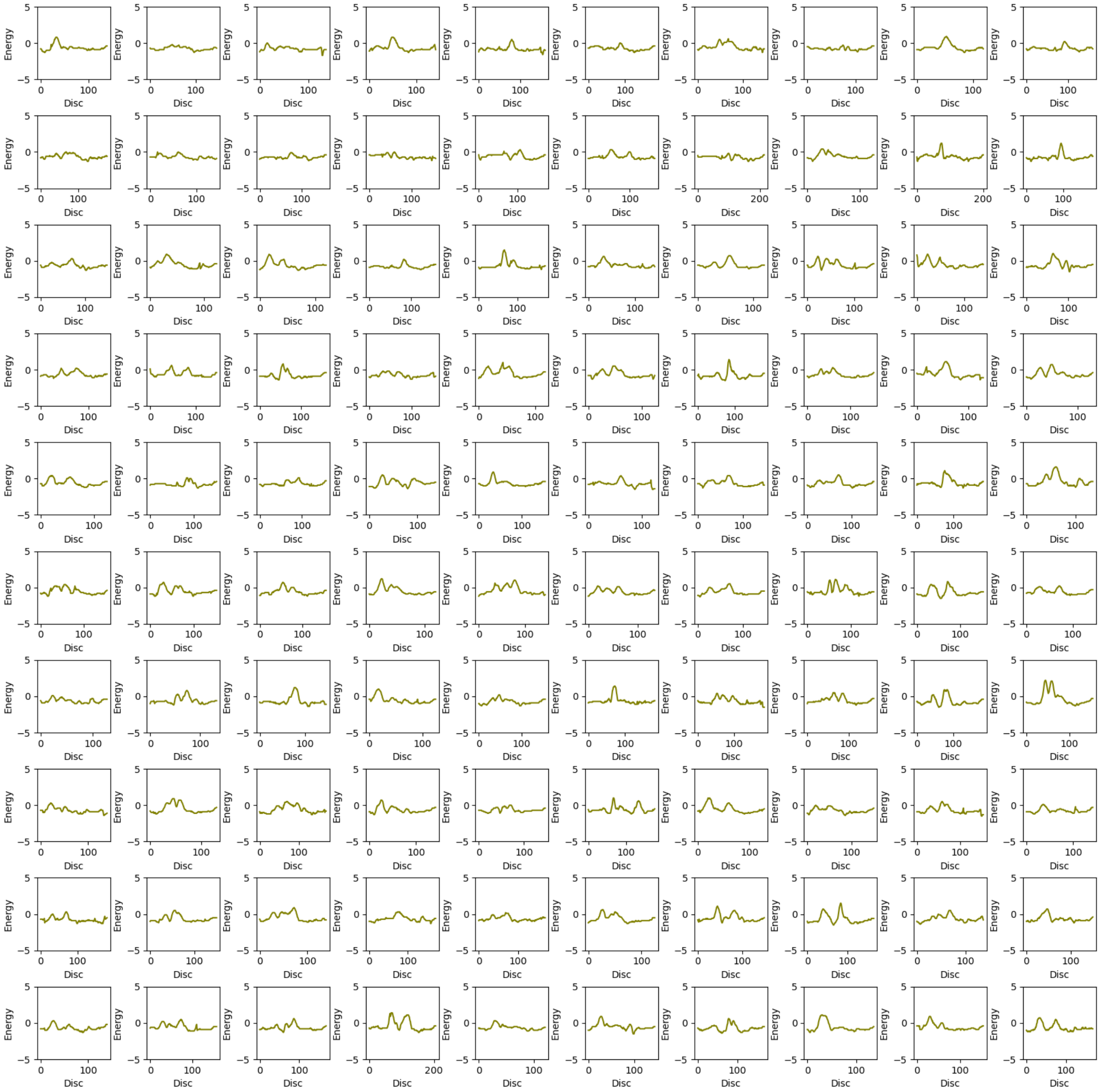

Supplement: Supplementary file 2 — ci4c00966_si_002.pdf [file ci4c00966_si_002.pdf]
